# Brazilian guidelines on diagnosis and management of traumatic vascular injuries

**DOI:** 10.1590/1677-5449.202300422

**Published:** 2023-10-30

**Authors:** Adenauer Marinho de Oliveira Góes, José Gustavo Parreira, Gustavo Henrique Dumont Kleinsorge, Marcelo Bellini Dalio, Pedro Henrique Ferreira Alves, Francisco João Sahagoff de Deus Vieira Gomes, Walter Junior Boim de Araujo, Edwaldo Edner Joviliano, Julio Cesar Peclat de Oliveira

**Affiliations:** 1 Sociedade Brasileira de Angiologia e de Cirurgia Vascular - SBAC, São Paulo, SP, Brasil.; 2 Sociedade Brasileira de Atendimento Integrado ao Traumatizado - SBAIT, São Paulo, SP, Brasil.; 3 Centro Universitário do Pará - CESUPA, Faculdade de Medicina, Belém, PA, Brasil.; 4 Universidade Federal do Pará - UFPA, Faculdade de Medicina, Belém, PA, Brasil.; 5 Faculdade de Ciências Médicas da Santa Casa de São Paulo, Departamento de Cirurgia, São Paulo, SP, Brasil.; 6 Fundação Hospitalar do Estado de Minas Gerais - FHEMIG, Clínica de Cirurgia Vascular, Hospital João XXIII, Belo Horizonte, MG, Brasil.; 7 Universidade de São Paulo - USP, Divisão de Cirurgia Vascular e Endovascular, Hospital das Clínicas, Ribeirão Preto, SP, Brasil.; 8 Universidade de São Paulo - USP, Faculdade de Medicina, Hospital das Clínicas, III Clínica Cirúrgica, São Paulo, SP, Brasil.; 9 Universidade do Estado do Rio de Janeiro - UERJ, Faculdade de Ciências Médicas, Departamento de Cirurgia, Rio de Janeiro, RJ, Brasil.; 10 Polícia Militar do Estado do Rio de Janeiro - PMERJ, Rio de Janeiro, RJ, Brasil.; 11 Universidade Federal do Paraná - UFPR, Hospital das Clínicas, Divisão de Angiorradiologia e Cirurgia Endovascular, Curitiba, PR, Brasil.; 12 Universidade Federal do Estado do Rio de Janeiro - UNIRIO, Departamento de Cirurgia, Rio de Janeiro, RJ, Brasil.

**Keywords:** wounds and injuries, vascular system injuries, blood vessels, guidelines as topic

## Abstract

Trauma is a leading cause of death, permanent disability, and health care cost worldwide. The young and economically active are the most affected population. Exsanguination due to noncompressible torso hemorrhage is one of the most frequent causes of early death, posing a significant challenge to trauma and vascular surgeons. The possibility of limb loss due to vascular injuries must also be considered. In recent decades, the approach to vascular injuries has been significantly modified. Angiotomography has become the standard method for diagnosis, endovascular techniques are currently incorporated in treatment, and damage control, such as temporary shunts, is now the preferred approach for the patients sustaining physiological derangement. Despite the importance of this topic, few papers in the Brazilian literature have offered guidelines on vascular trauma. The Brazilian Society of Angiology and Vascular Surgery has developed *Projetos Diretrizes* (Guideline Projects), which includes this publication on vascular trauma. Since treating trauma patients is a multidisciplinary effort, the Brazilian Trauma Society (SBAIT) was invited to participate in this project. Members of both societies reviewed the literature on vascular trauma management and together wrote these guidelines on vascular injuries of neck, thorax, abdomen, and extremities.

## INTRODUCTION

Vascular injury is an important cause of morbidity and mortality. Current studies show that the occurrence of these injuries has progressively increased over time; according to current estimates, about 6% of civilian trauma victims have some vascular injury.^1-3^

In recent decades, the diagnosis and treatment of vascular trauma has undergone significant changes, such as recommended use of tourniquets in the pre-hospital environment, the replacement of traditional angiography with angiotomography as the first choice for evaluating injuries, the use of damage control protocols such as temporary vascular shunts, and the increasingly frequent use of endovascular techniques for temporary control or definitive treatment of bleeding.^1,2,4-9^ Despite these advances, mortality continues to increase in trauma patients. Trauma currently kills more patients under 50 years of age than oncological or cardiovascular diseases, overloading the health system, and hemorrhaging associated with vascular injury is a challenge for surgeons.^3,5^

At an American level 1 trauma center, the number requests for urgent evaluations by vascular surgeons has increased by about 500% in the last 15 years, with vascular surgeons being more frequently called upon to deal with ischemic limbs, active bleeding, and complex surgeries.^3^ One reason for the growing demand of vascular surgeons in trauma services is that general/trauma surgeons receive less and less training in vascular surgery, which has a negative impact on their ability to repair trauma injuries in blood vessels.^3^

The epidemiological peculiarities of vascular trauma vary according to country. In Europe, Japan and Australia, blunt trauma predominates, such as falls from heights and traffic accidents, whereas in the United States, Africa, and South America, penetrating injuries predominate.^1^ Brazilian data confirm that gunshot and stab wounds are the most common type of vascular injury in the country, affecting mainly the extremities in young men.^10-12^

In 2022, the board of directors of the Brazilian Society of Angiology and Vascular Surgery (SBACV) determined that the Society’s guidelines on various topics should be updated; however, no previous guidelines had considered the Brazilian context of vascular trauma presentation and treatment resources. Given that many vascular injuries are treated by surgeons who are not specialists in vascular surgery, the Brazilian Trauma Society (SBAIT) was invited to join the guideline working group.

The objective of these guidelines is to standardize the approach to vascular trauma in different body regions, emphasizing diagnosis and treatment according to the best evidence and the consensus of a group of surgeons experienced in vascular injuries.

## METHODS

The boards of SBACV and SBAIT formed a working group of 6 surgeons experienced in vascular trauma treatment. One member acted as project coordinator.

The members were assigned questions about diagnostic and therapeutic strategies for vascular trauma in the neck, chest, abdomen, and limbs. Each participant then investigated the questions based on the vascular trauma literature, searching the MEDLINE, SciELO, LILACS, Scopus, and Embase databases. The best available evidence was considered. The descriptors included: aorta, artery, axillary, brachial, cava, diagnosis, femoral, fibular, iliac, injury, popliteal, radial, subclavian, tibial, trauma, treatment, ulnar, and vein. Articles published between 1990 and 2022 in Portuguese or English were included.

The coordinator did not answer questions but received the responses of each participant; the participants had no access to the responses of their peers. Based on the responses, the coordinator formulated recommendations on the diagnosis and treatment of vascular injuries in each body region and sent them to the participants in rounds of email. Each recommendation was scored from 1 to 5 according to the degree of agreement. For scores ≤ 3, the participants were asked to explain the discrepancy and propose modifications based on specific references in the literature. Using the Delphi method,^13^ the coordinator modified each recommendation until it received a minimum score of 4, reflecting a high degree of agreement (80%) among the participants.

Table 1 shows what were considered hard and soft signs of vascular trauma. Abdominal and limb injury classifications are shown in Tables 2 and 3, respectively, in accordance with the recommendations of the American Association for the Surgery of Trauma (AAST).^1,2^

**Table 1:** Clinical signs of peripheral vascular injury.

**Hard signs**	**Soft signs**
Pulsatile bleeding	Non-pulsatile bleeding
Pulsating or expanding hematoma	Non-pulsatile or non-expanding hematoma
Loss of distal pulse	Reduced pulse
Murmur and/or thrill	History of arterial bleeding (massive)
Hypotension	Previous tourniquet
	Neurological deficit
	Injury in the path of the trunk vessels

Adapted from Kobayashi et al.^2^

**Table 2:** AAST grading scale for abdominal vascular trauma injuries.

**Degree**	**Injury**
I	Innominate mesenteric venous arterial and/or tributary branches
	Phrenic artery and/or vein
	Lumbar artery and/or vein
	Gonadal artery and/or vein
	Ovarian artery and/or vein
	Other small artery and/or brachiocephalic vein that requires ligation
II	Right, left, or common hepatic artery
	Splenic artery or vein
	Right or left gastric artery
	Gastroduodenal artery
	Inferior mesenteric artery or vein
	Named primary branches of the superior mesenteric artery or tributaries of the superior mesenteric vein (eg, ileocolic artery)
	Other abdominal vessels that require ligation or repair
III	Trunk of the superior mesenteric vein
	Renal artery and/or vein
	Iliac artery and/or vein
	Hypogastric artery and/or vein
	Infrarenal inferior vena cava
IV	Trunk of the superior mesenteric artery
	Celiac trunk
	Suprarenal or infrahepatic inferior vena cava
	Infrarenal abdominal aorta
V	Portal vein
	Extraparenchymal hepatic vein
	Retrohepatic or suprahepatic inferior vena cava
	Subdiaphragmatic suprarenal abdominal aorta

AAST = American Association for the Surgery of Trauma. Adapted from Kobayashi et al.^1^

**Table 3:** AAST grading scale for traumatic peripheral vascular injuries

**Degree**	**Injury**
I	Artery/Digital vein
	Artery/Palmar vein
	Artery/Deep palmar vein
	Dorsal pedal artery
	Artery/Plantar vein
	Innominate venous arterial/tributary branches
II	Basilic/cephalic vein
	Saphenous vein
	Radial artery
	Ulnar artery
III	Axillary vein
	Superficial/deep femoral vein
	Popliteal vein
	Brachial artery
	Anterior tibial artery
	Posterior tibial artery
	Fibular artery
	Tibiofibular trunk
IV	Superficial/deep femoral artery
	Popliteal artery
V	Axillary artery
	Common femoral artery

AAST = American Association for the Surgery of Trauma. Adapted from Kobayashi et al.^2^

Denver (modified Biffl) classifications were used for recommendations on blunt cervical vascular injuries (Table 4),^14^ while Society for Vascular Surgery classifications were used for blunt trauma to the thoracic aorta (Table 5).^15^

**Table 4:** Denver grading scale for blunt carotid arterial injuries.

Grade I	Arteriographic image of vessel wall irregularity or intramural dissection/hematoma with luminal stenosis < 25%.
Grade II	Intraluminal thrombus or raised intimal flap is visualized, or intramural dissection/hematoma with associated luminal narrowing ≥ 25%.
Grade III	Pseudoaneurysm.
Grade IV	Occlusion.
Grade V	Transection with free extravasation of contrast material.

Adapted from Biffl et al.^14^

**Table 5:** Classification system for traumatic injuries of the thoracic aorta.

**Grade**	**Injury**
I	Intimal tear
II	Intramural hematoma
III	Pseudoaneurysm
IV	Rupture

Adapted from Lee et al.^15^

The levels of evidence were ranked according to the GRADE system,^16^ which has been previously used in international guidelines for abdominal^1^ and peripheral vascular injuries^2^ published by the American Association for the Surgery of Trauma and the World Society of Emergency Surgery (Table 6).

**Table 6:** Modified GRADE system for strength of recommendation.

**Degree of recommendation**	**Clarity of risks/benefits**	**Quality of supporting evidence**	**Implications for clinical practice**
1A	The benefits clearly outweigh the risks and burdens, or vice versa.	Consistent evidence from well-conducted, randomized controlled trials, or overwhelming evidence in some other form. Further research is unlikely to change our confidence in the benefit and risk estimate.	Strong recommendations that can be applied to most patients in most circumstances without reservations. Physicians should follow a strong recommendation unless there is a clear rationale for an alternative approach.
Strong recommendation. High evidence quality.			
1B	The benefits clearly outweigh the risks and burdens, or vice versa.	Evidence from randomized controlled trials with important limitations (inconsistent results, methodological flaws, or indirect/imprecise measurements) or very strong evidence from some other research design. New research may impact our confidence in the benefit and risk estimates.	Strong recommendation that applies to most patients. Physicians should follow a strong recommendation unless there is a clear and compelling rationale for an alternative approach.
Strong recommendation. Moderate evidence quality.			
1C	The benefits seem to outweigh the risks and burdens, or vice versa.	Evidence from observational studies, unsystematic clinical experience, or randomized controlled trials with serious flaws. Any effect estimates are uncertain.	Strong recommendation that applies to most patients. However, some of the evidence base supporting the recommendation is of poor quality.
Strong recommendation. Low evidence quality.			
2A	Benefits are closely balanced with risks and burdens.	Consistent evidence from well-conducted, randomized controlled trials, or overwhelming evidence in some other form. Further research is unlikely to change our confidence in the benefit and risk estimates.	Weak recommendation. The best course of action may differ depending on the circumstances or the values of patients or society.
Weak recommendation. High evidence quality.			
2B	Benefits are closely balanced with risks and burdens; some uncertainty in estimates of the benefits, risks, and burdens.	Evidence from randomized controlled trials with important limitations (inconsistent results, methodological flaws, or indirect/imprecise measurements), or strong evidence from some other research design. New research may change the benefit and risk estimates.	Weak recommendation. Alternative approaches are likely to be better for some patients in some circumstances.
Weak recommendation. Moderate evidence quality.			
2C	Uncertainty in estimates of benefits, risks, and burdens; benefits may be closely balanced with risks and burdens.	Evidence from observational studies, unsystematic clinical experience, or from randomized controlled trials with serious flaws. Any effect estimates are uncertain.	Weak recommendation. Other alternatives may be reasonable.
Weak recommendation. Low evidence quality.			

Adapted from Kobayashi et al.^1^

The final text was reviewed by the group and considered a structured consensus.

## QUESTIONS AND RECOMMENDATIONS

### General recommendations on vascular trauma

#### When necessary, what is the vascular substitute of choice?

Recommendations:

- For most cervical and limb vessels, the graft of choice is autologous vein, preferably from an uninjured limb. The great saphenous vein is the most common source. (1C).
- Autologous veins are preferable for reconstructing vessels ≤ 7 mm in diameter. For larger caliber vessels, such as large thoracic and abdominal vessels, vascular prosthesis is the most common solution. (1C).
- If no autologous vein is available (in cases of incompatible caliber or when the surgery cannot be prolonged for graft retrieval), polytetrafluoroethylene or Dacron prostheses can be used. Polytetrafluoroethylene has a lower incidence of infectious complications (1C).
- When the abdominal cavity has been contaminated, the use of prostheses should be avoided if possible (2C).
- Although extensive injuries in the iliac veins and inferior vena cava are often treated with ligation, grafting with “coiled” great saphenous vein has been performed in selected cases. (1C).^1,2,17-20^

#### What are the indications for damage control in vascular trauma and how is it performed?

Recommendations:

- Indications for damage control include:
  - Patients with coagulopathy, metabolic acidosis (base excess ≤ 10 or pH < 7.1) and hypothermia (lethal triad) in need of vascular reconstruction (1C).
  - Concomitant injuries requiring immediate treatment (1C).
  - Patients *in extremis* (1B).
  - Patients who need multiple transfusions of blood products (1B).
  - When the necessary resources for definitive treatment of the vascular injury are unavailable (1B).
- The most frequently employed techniques for damage control in vascular trauma are:
  - Ligation of non-essential vessels (2C).
  - Temporary vascular shunt implantation in vessels whose flow must be preserved (2C).
  - Temporary hemostasis by tamponade with balloons or compresses (2C).
  - Damage control techniques for associated non-vascular injuries, such as laparotomy in abdominal trauma and abbreviated synthesis of anatomical planes in other body regions.^1,2,5,17,21^

#### Is perioperative anticoagulation and/or antiplatelet aggregation indicated in traumatic vascular injuries? If so, how is it performed?

Recommendations:

- In blunt cervical trauma, grade I and II injuries of the carotid and vertebral arteries can be managed exclusively with antiplatelet aggregation and/or anticoagulation (1C). Although no evidence could be found in favor of a particular long-term antiplatelet aggregation or anticoagulation regimen, anticoagulation with heparin (preferably unfractionated) is initially recommended due to the possibility of reversal if bleeding complications occur (2C). No data could be found to support the use of anticoagulation or antiplatelet aggregation after carotid reconstruction.
- Although there is no evidence that anticoagulation and/or antiplatelet aggregation should be routinely recommended after surgical treatment of thoracic vascular injuries, both can be used in non-operative treatment of minor injuries in the subclavian and brachiocephalic arteries (2C). No specific recommendations were found regarding medication, dosage, or duration for such situations.
- In abdominal trauma, if vascular repair promotes significant venous stenosis, venous ligation is recommended. When the vein in question cannot be ligated (eg, inferior vena cava cranial to the renal veins), anticoagulation should be considered and, in such cases, there is no consensus regarding dosage or duration. Patients with abdominal/pelvic venous injuries should be monitored for venous thromboembolisms (2C).
- There is no evidence to support the use of intraoperative systemic anticoagulation or postoperative antiplatelet aggregation/anticoagulation in most vascular repairs in limbs. The exception is prolonged ischemic time, including occlusion of smaller vessels (2C).
- Every vascular injury patient in bed should receive prophylactic heparin to prevent venous thromboembolisms, although contraindications for anticoagulation must be considered (2C).^1,2,16,22-26^

### Recommendations on cervical vascular trauma

#### When should imaging exams be used to diagnose cervical vascular injury?

Recommendations:

- In patients with penetrating neck trauma who show no hard signs of vascular trauma and in patients with blunt trauma with a compatible mechanism* and cervical vascular trauma (1C).
  - Modified Denver criteria: cervical spine/face/skull base fractures, diffuse axonal injury with a Glasgow Coma Scale score < 6, and a trauma mechanism suggestive of vascular trauma (direct cervical impact, strain, or rotation) (2C).^23,24,26 27^

#### Which imaging tests should be performed?

Recommendations:

- computed tomography (CT) angiography is suitable for evaluating injuries in cervical zones 1, 2 and 3. Zone 2 injuries can also be evaluated by ultrasound (1C).^8,17,19^

#### What is the role of endovascular treatment in cervical vascular trauma?

Recommendations:

- Cervicotomy remains the preferred option for injuries with less complex surgical access, such as carotid injuries in cervical zone 2 (1C).
- The main indications for endovascular treatment are: (a) blunt or penetrating carotid trauma in areas with difficult surgical access (zones 1 and 3) and (b) penetrating trauma to the vertebral artery (embolization of the non-dominant artery and implantation of a coated stent in the dominant artery) (1C).
- Devices intended for vascular occlusion (embolization) or vascular reconstruction (coated stents) can be used in different injury patterns (eg, pseudoaneurysms, arteriovenous fistulas, and partial or total sections of the vessel) resulting from blunt or penetrating mechanisms (1C).^28-30^

#### Which cervical vascular injuries require watchful waiting?

Recommendations:

- Blunt trauma: patients without neurological symptoms who have grade I carotid injuries (intimal injury with < 25% luminal reduction), grade II (dissection or hematoma with more than 25% luminal reduction) or grade IV (occlusion) injuries, or vertebral artery injuries (1C).
- Penetrating trauma: patients without neurological symptoms who have carotid or vertebral artery occlusion; finding minimal vascular injuries on imaging (a non-obstructive intimal flap or pseudoaneurysm < 5 mm in diameter) (1C); and venous injuries without active bleeding (2C), provided there is no concomitant indication for cervicotomy due to associated injuries.^8,24,25^

#### Which cervical vascular injuries can be treated by ligation?

Recommendations:

- All venous injuries except bilateral injuries to the internal jugular veins. Since bilateral ligation is associated with intracranial hypertension, reconstructing at least 1 internal jugular vein is recommended (1C).
- Injuries to the external carotid arteries and their branches, as well as to vertebral arteries, can be treated by ligation (1C).
- Internal carotid injuries in zone 3, when surgical field limitations preclude temporary shunt implantation or vascular reconstruction (2C).
- Carotid injuries in patients *in extremis*, when it is impossible to place a temporary vascular shunt (2C).^28,31,32^

#### Is the use of a shunt recommended while correcting carotid injuries?

Recommendations:

- There is no evidence that using a shunt in more complex reconstructions (such as interposition of venous grafts) significantly affects neurological outcomes (2C).
- The use of shunts is especially recommended in cases of inadequate reflux or when carotid reconstruction cannot be completed in the first operation (damage control)(2C).^33,34^

#### When the carotid artery is thrombosed after penetrating trauma, should it be revascularized?

Recommendations:

- If the patient’s Glasgow Coma Scale score is < 8, there is a minimal likelihood that carotid reconstruction will be beneficial, and ligation may be performed (2C).
- If the patient’s Glasgow Coma Scale score is > 8, reconstruction of the carotid artery should be attempted unless there are contraindications for repair in penetrating injuries, eg, inaccessible injuries, coma > 4 h, large areas of cerebral infarction in imaging on admission, or lack of reflux in the distal arterial segment after thrombectomy (2C).^17,18,32^
- Note: For guidance on managing carotid thrombosis after blunt trauma, see recommendation 1.3.

### Recommendations on thoracic vascular trauma

#### When is thoracotomy indicated if imaging tests are not performed?

Recommendations:

- Victims of thoracic trauma who have persistent hemodynamic instability after initial trauma care (including pneumothorax treatment) or significant hemorrhaging through pleural drainage (> 1200 mL or persistent drainage of 200 mL/h) (1C).^17,21,35-37^

#### When should imaging tests be used to diagnose thoracic vascular injury?

Recommendations:

- Symptomatic patients should be actively investigated if hemodynamic conditions permit it (1C).
- Asymptomatic patients should be investigated whenever the trauma mechanism is compatible with vascular injury, for example:
  - Penetrating wounds to a vascular path or mediastinal transfixion.
  - Blunt trauma with a mechanism of injury is suggestive of vascular injury (eg, significant deceleration in a fall from a height, traffic accidents, or fractures of the scapula, clavicle, or first costal arch)(1C).
  - Polytrauma with a Glasgow Coma Scale score < 12 or severe traumatic brain injury (1C).^17,35,36,38,39^

#### Which imaging tests should be performed?

Recommendations:

- Unstable patients can undergo chest radiography in the emergency room and extended focused abdominal sonography for trauma (e-FAST) (1C).
- For stable patients, chest CT angiography is the examination of choice (1C).
- Angiography can be performed to resolve persistent questions, but this method is more commonly an endovascular therapeutic procedure (1C).^17,35,36,40,41^

#### Which thoracic vascular injuries require watchful waiting?

Recommendations:

- Minimal injuries to the subclavian artery (small dissection/pseudoaneurysm) can be monitored non-operatively, as well as blunt trauma that causes occlusion in the subclavian artery, provided adequate limb perfusion. In such cases, clinical and radiological follow-up is recommended (2C).
- Minimal blunt aortic injuries (grades I and II) may be amenable to non-operative treatment; beta-blockers and blood pressure control can be used in hemodynamically stable patients (1C)^17,35,37,38,41-44^ .

#### What is the role of endovascular treatment in vascular trauma in thoracic trauma?

Recommendations:

- Proximal control can be obtained in some injuries by through balloon catheters inserted using endoclamp techniques, which reduces bleeding and facilitates dissection of the traumatized vessel (1C).
- Endovascular treatment is preferable for grade III (pseudoaneurysm) and IV (rupture) blunt trauma to the aorta (1C).
- Endovascular treatment can be a first choice for blunt and penetrating trauma to the subclavian and axillary arteries, even in unstable patients, provided the necessary resources are available and a thoracotomy is not a treatment priority (1C). Although infrequent, treatment of trauma to the brachiocephalic trunk and vein has been reported (2C). In addition to material and logistical resources, the team must be experienced in endovascular treatment of these injuries.^35,37,38,42,43,45^

#### Which vessels can be ligated as a definitive treatment?

Recommendations:

- Veins: Almost all thoracic veins can be ligated without major consequences. The exceptions are the superior vena cava and the intrapericardial segment of the inferior vena cava. The internal jugular can be ligated, but bilateral ligatures should be avoided (2C).
- Arteries: subclavian arteries can be ligated proximally to the origin of the vertebral artery; under these conditions, upper limb perfusion can be maintained through the “subclavian steal” phenomenon (1C). In massive hemothorax and emergency thoracotomy, pulmonary artery ligation requires pneumonectomy (2C).^17,21,35,44^

#### Which vessels should be reconstructed whenever possible?

Recommendations:

- Veins: superior vena cava, the intrapericardial segment of the inferior vena cava, and at least one internal jugular vein (in case of bilateral injuries) (1C).
- Arteries: ligation of the thoracic arteries is an exceptional situation; flow in the aorta, brachiocephalic trunk, and intrathoracic segments of the common carotid arteries should be maintained whenever possible (1C).^17,21,46^

#### Which access routes are used for thoracic vascular injuries?

Recommendations:

- In stable patients, imaging tests can help with planning. The most frequent access routes and their structures include (1C):
  - Median sternotomy: the heart, ascending aorta, aortic arch, pulmonary arteries, brachiocephalic trunk and vein, superior vena cava, and intrapericardial segment of the inferior vena cava.
  - Median sternotomy and cervical or supraclavicular extension: the arterial brachiocephalic trunk and proximal subclavian arteries and veins. High left anterolateral thoracotomy (second/third intercostal space) to access the proximal segment of the left subclavian artery has also been described.
  - Left posterolateral thoracotomy in the fourth/fifth intercostal space: descending thoracic aorta, left pulmonary hilum and pulmonary veins.
  - Left anterolateral thoracotomy in the fourth/fifth intercostal space: left pulmonary hilum and heart.
  - Left anterolateral thoracotomy in the fourth/fifth intercostal space: left pulmonary hilum and heart.
  - Supraclavicular and/or infraclavicular (with or without clavicle excision): subclavian and axillary arteries and veins.
- In unstable patients, there is not enough time for imaging tests, and surgical access must be determined according to the clinical presentation:
  - Left anterolateral thoracotomy at the fourth/fifth intercostal space: resuscitation thoracotomy.
  - If the trauma affects both sides of the thorax, exploration can be initiated by an anterolateral thoracotomy in the fourth/fifth intercostal space on the side in which chest drain output shows greater hemothorax.
  - Bilateral thoracotomy (clamshell incision) in the fourth/fifth intercostal space is the only route that allows simultaneous access to the mediastinum and the 2 pleuropulmonary cavities; it can be used in unstable patients when the injury’s topography is unknown.^17,21,37,47^

### Recommendations on abdominal vascular trauma

#### When can laparotomy be indicated without imaging tests?

Recommendations:

- Hemodynamically unstable patients with a suspected abdominal vascular injury should undergo surgery without prior imaging (1B). Hemodynamic instability is secondary to penetrating abdominal trauma or blunt trauma with hemoperitoneum detected by FAST or peritoneal lavage. These indications are related to intra-abdominal vascular injuries and do not exclude other indications for laparotomy, such as clinical signs of peritonitis and evisceration.^1,22,48^

#### When should imaging tests be used to diagnose vascular injury? Which examinations should be ordered?

Recommendations:

- Hemodynamically stable patients with signs of intra-abdominal injury/mechanism compatible with vascular injuries*. However, if immediate laparotomy is not indicated, they should undergo angiotomography to screen for vascular injuries and plan surgical access (1A). Trauma mechanisms compatible with vascular injuries include pelvic fractures (mainly unstable), lumbar spine fractures, rapid deceleration mechanism, or seat belt sign.
- CT angiography is the recommended examination for diagnosing abdominal vascular injuries (1A).
- Ultrasound should be used to screen for intra-abdominal fluid, but not to diagnose abdominal vascular injuries due to its low accuracy for this purpose (1B).
  Note: In the current context, angiography is mainly used in therapeutic endovascular procedures.^1,49-51^

#### Which injuries can be treated non-operatively?

Recommendations:

- Non-operative treatment can be considered in stable patients with isolated injuries (AAST grade I-III) and no contrast extravasation on angiotomography (2C).

Note 1: The literature also reports cases of blunt and penetrating injuries to the inferior vena cava (1C), abdominal aorta (C2), portal vein (1C), superior mesenteric artery, and celiac trunk that were treated non-operatively.

Note 2: Before considering non-operative treatment, it is essential to assess hemodynamic stability and the possibility of associated non-vascular injuries.

Note 3: The development of institutional protocols for non-operative treatment in abdominal injuries is strongly recommended.^1,5,10,22,52-55^

#### What is the role of endovascular treatment in abdominal vascular trauma?

Recommendations:

- Endovascular treatment may be a definitive treatment option for stable patients with isolated injuries (AAST grade I-IV) (2C).
- Resuscitative Endovascular Balloon Occlusion of the Aorta (REBOA) can be used as part of resuscitation maneuvers until definitive treatment can be performed (2C).
  Note: according to the literature, endovascular techniques can be used for the following injuries/situations: injuries to the superior mesenteric artery (2C), thrombosis of the renal artery due to blunt trauma (2C), embolization of the internal iliac artery and its branches in the context of pelvic fractures (2C), and blunt and penetrating trauma to the inferior vena cava and abdominal aorta (2C). Endovascular balloon occlusion to control bleeding in association with open surgical procedures (endoclamp) for trauma in difficult-to-access segments has also been described, including the retrohepatic and suprahepatic segments of the inferior vena cava (2C).^1,5,17,48,53,56^

#### During a laparotomy, when should retroperitoneal hematomas be explored? What vascular exposure/control maneuvers are recommended?

Recommendations:

- Retroperitoneal hematomas associated with penetrating trauma must be explored, regardless of the affected area (1B). Penetrating injuries of the retrohepatic cava can be an exception to this rule, provided that the hematomas are contained and the patient is stable. Non-operative treatment has even been described in certain cases.
- Zone 1 retroperitoneal hematomas associated with blunt trauma should be explored (1B).
- Zone 2 hematomas associated with blunt trauma should be explored if they show signs of expansion or pulsatility (1B).
- Stable zone 3 hematomas associated with blunt trauma should not be explored (1B). Exceptions to this rule include the lack of a femoral pulse and cases in which radiological evaluation/surgical exploration of the common and external iliac arteries must be performed to rule out injury. During surgical exploration, medial advancement should be avoided to reduce the risk of bleeding associated with pelvic hematoma removal.
  Vascular exposure/control maneuvers related to the exploration of retroperitoneal hematomas (1B):
    - Supraceliac clamping of the aorta in the abdominal cavity behind the greater omentum at the level of the diaphragmatic hiatus is indicated for proximal control of the aorta in the left diaphragmatic crura in cases of large retroperitoneal hematomas.
    - The Mattox maneuver (medial visceral rotation after incising the parietal peritoneum at the left paracolic gutter): to expose the left side of zone 2 and the infrarenal segment of the aorta and left iliac vessels, a modified Mattox maneuver can be performed, which does not include mobilizing the left kidney. The classic maneuver, which rotates the kidney, allows exposure of the entire length of the abdominal aorta and the origin of its visceral branches.
    - The Cattell-Braasch maneuver (medial visceral rotation by incising the parietal peritoneum at the right paracolic gutter) allows exposure of the right iliac vessels and the infrarenal portion of the inferior vena cava. It can be complemented with the Kocher maneuver, which, through duodenopancreatic mobilization, exposes the perirenal segment of the cava to the lower border of the liver.
    - Anterior transperitoneal access: incising the retroperitoneum longitudinally between the duodenum and the inferior mesenteric vein allows access to the infrarenal portion of the vena cava and aorta.
    - The Pringle maneuver consists of clamping the portal triad. Although it does not expose the retroperitoneum, in the context of vascular injuries, it is often used as part of the necessary maneuvers to approach retrohepatic cava injuries.
    - To expose the portal vein and retropancreatic superior mesenteric vein in patients with major active bleeding, sectioning the pancreas (usually already injured) may be necessary. If necessary, a caudal pancreatectomy and splenectomy should be performed.^1,17,48^

#### Which vessels can be ligated as a definitive treatment?

Recommendations:

- Veins:
- The common, external and internal iliac, inferior mesenteric, and splenic veins (if the splenic vein is ligated, a splenectomy must be performed) can be ligated as a definitive treatment (1C).
- When performing damage control, the infrarenal vena cava, superior mesenteric vein and portal vein can be ligated (1C). If the superior mesenteric or portal vein is ligated, laparostomy is indicated; special attention must be paid to volume replacement to compensate for the volume trapped in the splanchnic system. The portal vein should only be ligated if the hepatic artery is intact. At least one of these structures must remain patent to maintain hepatic perfusion (1C).
- Renal veins: to maintain venous drainage, the left renal vein should preferably be ligated close to the opening of the inferior vena cava. If the right renal vein is ligated, a right nephrectomy should be performed (1C).
- Arteries:
- The celiac trunk and its branches: consider cholecystectomy, since there are reports of gallbladder necrosis after hepatic artery ligation (the risk may be greater if the ligation is distal to the origin of the gastroduodenal artery). If the splenic artery ligation is proximal, splenectomy may not be necessary (1C).
- Superior mesenteric artery: ligate distal to the origin of the middle colic artery (Fullen zones III and IV). It will likely be necessary to perform a segmental enterectomy to treat the resulting ischemia (1C).
- Renal arteries: complement with nephrectomy.
- The inferior mesenteric artery and internal iliac arteries can be ligated (1C).^1,17,48,52,54,57-59^

#### Which vessels must be reconstructed?

Recommendations:

- When reconstruction is impossible, the flow of the following vessels should be preserved whenever possible. In the context of damage control, implanting a temporary vascular shunt is suggested for:
  - The suprarenal segment of the inferior vena cava (1C).
  - The aorta and common and external iliac arteries (1C).
  - Proximal segments of the superior mesenteric artery (Fullen 1 and 2) (1C).
  - The patency of the portal vein or hepatic artery must be maintained to ensure hepatic perfusion (1C).^1,17,48,52,54,57-59^

#### What is the procedure for injuries associated with high contamination or a risk of digestive fistula?

Recommendations:

- Ligatable vessels should be ligated.
- If a graft is required, autologous veins are preferred. Prostheses can be used to reconstruct large-caliber vessels. If possible, the prostheses should be kept “protected” from contact with the viscera, preferably by interposing the retroperitoneum (1C).
- Vascular ligation associated with reconstruction involving an extra-anatomical bypass can also be performed in regions with a lower risk of infection (1C).^1,17^

#### When should lower limb fasciotomy be considered in abdominal vascular injuries?

Recommendations:

- Injuries to the aorta and common or external iliac arteries that cause prolonged ischemia in the lower limbs may result in compartment syndrome after lower limb reperfusion (1C).
- Ligation of the inferior vena cava can cause significant edema in the lower limbs and compartment syndrome (1C).
  In both situations, the lower limbs should be monitored and, if necessary, a fasciotomy should be performed. A prophylactic fasciotomy is not mandatory.^1,17,55^

#### What is the treatment sequence for hematomas associated with hip fractures?

Recommendations:

- Unstable hip fractures should be identified during physical examination in the primary assessment of trauma patients. Femoral pulse symmetry should be assessed, since it may be absent when the common and external iliac arteries are injured (1C).
- Hemodynamically stable patients should undergo angiotomography (1C).
- The first specific measure in hemodynamically unstable patients is to reduce pelvic volume by applying special straps or improvised materials, such as sheets (1C).
- If available, endovascular balloon occlusion of the aorta (REBOA) can be performed in inflation zone III (1C).
- If intraperitoneal hemorrhage is ruled out (by FAST, peritoneal lavage, or laparotomy), the next priority is to reduce bleeding from the pelvic retroperitoneum. The 3 most common techniques for this are preperitoneal packing with compresses, external fracture fixation, and angioembolization (1C).
- Preperitoneal tamponade effectively reduces hemorrhages associated with venous plexus injuries, arterial branches, or fracture foci. In unstable fractures, external fixation should be applied whenever possible. When these techniques are combined, angioembolization is only necessary in a minority of cases (1B).
- Angiography should be considered in patients with pelvic fractures when there is (1C):
  - Hemodynamic instability or evidence of active bleeding after excluding other hemorrhagic foci (this step may be repeated if the patient has undergone angiography previously).
  - Contrast extravasation in tomography, regardless of the hemodynamic condition.
  - Age > 60 years, regardless of angiotomography findings or hemodynamic status.
  - Hematomas with an estimated volume > 500 mL.
- The best sequence for or ideal combination of preperitoneal packing, external fixation, and angioembolization remains ill defined. However, we consider the following points relevant:
  - Each institution must develop multidisciplinary protocols (trauma surgery/vascular surgery/orthopedics and traumatology) that are adequate for their treatment context.
  - Hemodynamically unstable patients should preferably be resuscitated in a surgical center to control bleeding foci. The liberal use of preperitoneal packing is recommended, and this could even precede fracture fixation. Ideally, pelvic volume should be reduced using sheets or other specific devices while performing preperitoneal tamponade.
  - Early fracture fixation should be considered by the orthopedic team. It is usually indicated for “open-book” pelvic fractures or other types involving a large opening in the pelvic ring. However, this measure does not apply to all unstable fractures.
  - Preperitoneal packing should be considered in patients with unstable pelvic fractures, especially those with persistent hemodynamic instability or signs of active bleeding after pelvic immobilization/external fixation, or in those who are not candidates for external fixation due to the specific characteristics of the fracture. This is an important option in services without angiography for embolization.
  - Angioembolization should be considered in patients with suspected arterial bleeding, which is often associated with hemodynamic instability and metabolic acidosis on admission, large retroperitoneal hematomas, and signs of contrast extravasation on angiotomography. However, in unstable patients, external fixation and preperitoneal packing should not be delayed to perform angioembolization, which is more effective when the bleeding is of arterial origin.
  - Angiography can also be performed in patients who remain unstable after fracture fixation. If hemodynamic instability persists and the resources are not available for endovascular procedures, embolization can be performed by surgically exposing the internal iliac arteries and direct arterial puncture to inject an autologous clot, Gelfoam® paste, or other appropriate materials. Internal iliac arteries should not undergo surgical ligation.^1,51,56,60^

### Recommendations on vascular injuries of the limbs

#### When should imaging tests be used to diagnose vascular injury?

Recommendations:

- The most frequent indications are:
  - Hemodynamically stable patients with soft signs and/or an ankle-brachial index < 0.9 (1B).
  - In cases where endovascular treatment is being considered (1B).
- Given sufficient hemodynamic stability, imaging tests are recommended in the following situations:
  - Different traumas in the same extremity (even with hard signs) to clarify which one caused the vascular injury and whether there is more than one concomitant vascular injury (2B).
  - Knee dislocation, even without signs of vascular trauma (2B).^2,61^

#### Which imaging tests should be performed?

Recommendations:

- CT angiography is the gold standard for investigating vascular trauma in the limbs (1B).
- Catheter angiography is indicated in cases of suspected vasospasm, when angiotomography is unavailable or imaging is compromised by artifacts from metallic objects (such as bullets), or as an integral part of endovascular treatment (1B).
- Doppler ultrasound can be used, including the fast Doppler protocol, but with restrictions (1B).
- Antiresonance is little used. It is contraindicated when metal fragments are present or suspected (1B).^2,7,62-69^

#### Is it safe to discharge patients based on physical examination (including ankle-brachial index) alone?

Recommendations:

- In penetrating trauma, an ankle-brachial index or blood pressure index ≥ 0.9 is sufficient for hospital discharge without imaging investigation when there are no signs of vascular injury. Outpatient follow-up is recommended due to the possibility of late manifestations of minor injuries (1B).
- Because physical examinations are less sensitive in high-energy mechanisms of blunt trauma injury (such as knee dislocations), imaging assessment is recommended (1B).^2,61^

#### When should non-operative treatment be indicated?

Recommendations:

- Arterial injuries in the hand and forearm (AAST I and II) in hemodynamically stable patients with adequate distal perfusion and no active bleeding (1C).
- Isolated injuries in tibial/peroneal arteries when there is an intact trunk artery to the foot and no active bleeding or distal ischemia (1C).
- AAST grade III injuries when there is another intact artery in the limb, adequate distal perfusion, and no active bleeding (1C).
- Franz’ screening criteria (2011): injury < 5 mm, adhered intimal flap, intact distal circulation and no hemorrhaging (1C).^2,64,67,70^

#### Can venous injuries in the limbs be ligated or must they be reconstructed?

Recommendations:

- As a general rule, when the necessary repair can be performed using venorrhaphy, large-caliber veins (subclavian, axillary, common and superficial femoral veins, and popliteal veins) should be reconstructed, which generally results in a better functional prognosis for the limb. When injuries require more complex reconstruction, especially in the context of damage control, any limb vein can be ligated (1C).^2,70,71^

#### Should associated nerve and tendon injuries be reconstructed in the initial or subsequent surgical procedures?

Recommendations:

- Both are possible. If there is hemodynamic stability and the vascular trauma has already resolved, nerve and tendon injuries can be treated during the same surgery. Depending on the systemic condition of the patient, the characteristics of the injury, and the center’s resources, treatment for these injuries can be postponed (1C).^2^

#### What criteria should be used to indicate fasciotomies?

Recommendations:

- Trauma mechanism: trauma to an extremity with ischemia > 4-6 h or high-energy trauma to a forearm or leg (1C).
- Clinical presentation: pain in the region of the affected muscle compartment, tense edema in the muscle compartment, paresis, paresthesia, and no distal pulse (1C).
- Intracompartmental pressure > 30 mmHg (1C).
- Surgical findings/operations: large vein ligature, arterial reconstruction after > 6 h of ischemia, association of arterial and venous injuries (especially with venous ligation), and vascular injuries associated with fractures (1C).^2,70^
- Notes:
  - The literature review indicates that early fasciotomy decreases the incidence of amputation and infectious complications in traumatized limbs.
  - The surgeon’s judgment overrides compartmental pressure measurements and complementary exams.
  - These comments do not exclude other indications not directly related to vascular trauma, such as burns or crushing.

#### Which arterial injuries can be treated with ligation?

Recommendations:

- Forearm or leg arteries when collateral vessels maintain perfusion (1C).
- The subclavian and axillary arteries can be ligated in patients *in extremis*. In injuries of the subclavian arteries, when proximal to the origin of the vertebral artery, there is a higher probability of adequate perfusion in upper limbs through the “subclavian steal” phenomenon (1C).
- Brachial artery injuries, distal to the origin of the deep brachial artery, can be ligated with a low risk of distal ischemia (1C).^2,61,70^

#### Can brachial artery thrombosis, associated with a supracondylar fracture of the humerus and adequate perfusion, be treated conservatively in children?

Recommendations:

- Yes. Due to extensive collateral circulation and the proportionally smaller muscle mass of the upper limb, when there is a brachial artery thrombosis with adequate perfusion, the injury can be treated conservatively (even in children) with a low risk of late complications (1C).^72,73^

#### In vascular trauma associated with fractures, what is the most appropriate treatment sequence?

Recommendations:

- The priority is to stop active bleeding. Long bone fractures should preferably be fixed prior to definitive vascular repair. If limb perfusion is inadequate, a temporary vascular shunt should be considered prior to fracture fixation. (1C).^2,60,69^

#### Which clinical criteria/scenarios and scores indicate primary amputation?

Recommendations:

- Scores:
  - A Mangled Extremity Severity Score (MESS) > 7 is the most common indicator (1C).
  - The Popliteal Scoring Assessment for Vascular Extremity Injuries in Trauma (POPSAVEIT) is specific for popliteal artery injuries; patients scoring 3-5 points are at high risk of amputation (1C).
- Clinical criteria/scenarios:
  - Hemodynamic instability due to hemorrhaging in a mutilated extremity with multiple exposed fractures and no other way to control hemorrhaging.
  - Late presentation without sensitivity or motor activity (1C).
  - Extensive tissue loss and/or the impossibility of functional recovery (1C).
  - The decision for primary amputation is complex and may involve several surgical specialties, such as orthopedics, vascular surgery, trauma surgery and plastic surgery. Other scores, in addition to those mentioned above, have been described; all involve limitations and, although they can help in decision making, they do not replace the surgeon’s judgment.^2,74^

#### How many intact arteries (radial or ulnar) in the forearm are sufficient to maintain limb viability?

Recommendations:

- Usually just one. If distal perfusion is maintained by another artery, the injured vessel can be ligated (1C).^2,61^

#### How many intact arteries (anterior tibial, posterior, or fibular) in the leg are sufficient to maintain limb viability?

Recommendations:

- Having ≥ 2 patent arteries reduces the likelihood of amputation in infrapatellar injuries (1C).
- The integrity of an anterior or posterior tibial artery, with adequate distal perfusion, may be sufficient to maintain limb viability (1C).
  Note: in the context of vascular trauma, the literature did not explicitly state that the integrity of the intact fibular artery alone is sufficient for adequate limb perfusion.^2,70^

#### What is the role of endovascular treatment in limb injuries?

Recommendations:

- Injuries of the subclavian and/or axillary arteries can receive definitive treatment (coated stents) or treatment adjuvant to open surgery through proximal control with balloon catheters (endoclamps) (1C).
- Late correction of arteriovenous fistulas and pseudoaneurysms (1C).
- Embolization of femoral artery branches (especially the deep femoral artery) and infrapatellar arteries (1C).
- The gold standard for vascular injuries of the extremities is still open surgical repair. Endovascular techniques, even in unstable patients, have been described with increasing frequency, especially for injuries to subclavian and axillary vessels, due to the complexity of surgical access to these structures. Resource availability, including staff training, should be considered before resorting to this possibility (1C).^2,45,61^

## DISCUSSION

Although patients of all ages are subject to traumatic vascular injuries, the vast majority of cases involve young men.^1-5,7,18,23,35,45,56^

The distribution of injuries and etiologies is not uniform around the world. In European, Australian and Japanese trauma centers, blunt mechanisms (eg, traffic accidents) predominate, while in Brazil and the USA most vascular injuries result from penetrating trauma due to gun and knife violence, which more frequently affects the extremities.^1,17,66,75^ In addition to accidents and violence, patients of all ages are increasingly undergoing diagnostic and therapeutic procedures that can cause iatrogenic vascular trauma.^3,22,48,76^

These injuries, in addition to jeopardizing the viability of the limbs, represent an important cause of death, which results in most cases from non-compressible hemorrhaging associated with trauma to the trunk vessels.^5,41-43,51,52,56-59,61^

Over time, new technologies have revolutionized the diagnosis and treatment of vascular trauma. However, human and technological resources are not evenly distributed. The purpose of these guidelines is to help standardize and improve vascular trauma treatment in Brazil according to the best available evidence from updated literature, developing applicable recommendations for the national care context.

The last decade has witnessed a paradigm shift in prehospital trauma care. In up to 20% of cases, tourniquets are used for temporary control of exsanguinating hemorrhage in the extremities, resulting in a significant mortality reduction.^2,70^ Obviously, tourniquets are only justified for significant bleeding and, as soon as possible, they should be removed for specialized evaluation and, if applicable, revascularization of the limb.

There is consensus that patients who arrive at the hospital with hard signs of traumatic vascular injuries, especially those with active bleeding and hemodynamic instability, should immediately undergo interventions to stop the hemorrhage and, if possible, restore blood perfusion in the affected limb/organ.^17,19,21,28,35,36^

One important update involves patients without clinical signs of vascular injury in the limbs. Although a physical examination is less able to detect vascular trauma in cases of high-energy blunt trauma (such as knee dislocations), in penetrating trauma, which is the main mechanism of vascular trauma to the extremities, current evidence supports hospital discharge without imaging tests, provided that the physical examination results are normal (no hard or soft signs of vascular injury) and the ankle-brachial index is ≥ 0.9.^2,6,62,63,69,75^

Although this strategy has been determined safe, venous injuries, pseudoaneurysms, arteriovenous fistulas, and other minor vascular injuries may not be detected through physical examination. Physician experience is fundamental and, if it is deemed necessary, further testing should be performed. After discharge, the patient should be referred for outpatient evaluation due to the risk of late complications from initially undetected minor injuries.^2,63,66^

For all scenarios that require imaging tests, CT angiography is the current gold standard in vascular trauma diagnosis.^2,35,64,65^

Therapeutic indications and intervention techniques have also evolved. In selected cases, some injuries can be managed non-operatively. This is the case, for example, in brachial artery injuries associated with a supracondylar humeral fracture in children, resulting in adequate distal perfusion but no palpable pulse - a condition known as “pink pulseless hand”^72,73^ . Compression injuries of the retrohepatic vena cava may also be subject to watchful waiting,^10,55^ and criteria for non-intervention in limb injuries have been established.^2,64,67,70^

Endovascular treatment has become established in certain contexts, such as blunt trauma to the thoracic aorta,^15,43,77^ and is becoming more frequent in penetrating traumas requiring complex accesses, such as in axillary-subclavian injuries.^35,37,38,45,78^

Since damage control strategies, including modern resuscitation concepts, have helped increase survival in very severe injuries,^2,17,52,57,59^ we recommend damage control training for all surgeons likely to treat patients with traumatic vascular injuries. Certain new strategies require more complex materials and procedures, such as endovascular occlusion of the aorta,^1,4^ while others can save limbs and lives with a segment of nasogastric tubing. Temporary vascular shunting, which is still uncommon in Brazilian hospitals, is becoming a mandatory skill for surgeons who deal with these types of trauma.^2,17-19,21^

Trauma is unlike other conditions that affect the circulatory system. Cases involve heterogeneous mechanisms, severity, and clinical courses, which often make it impossible to adequately record a series of variables. These factors make it difficult to write guidelines based on evidence of high scientific quality, and many recommendations have been based on case series or observational studies.

## CONCLUSIONS

Although technological advances have, in part, revolutionized the diagnosis and treatment of vascular trauma, management of these challenging injuries continues to be based on the early interruption of bleeding and restoring blood flow to the affected limb/organ.

The surgeon’s common sense and experience remain fundamental and, although the present recommendations cannot replace them, we hope they can help standardize and strengthen vascular trauma treatment, especially considering the heterogeneity and particularities of care in our country.
