# Supplementary material for: Brazilian guidelines on diagnosis and management of traumatic vascular injuries
Source: J Vasc Bras. 2023 Oct 30;22:e20230042. doi: 10.1590/1677-5449.202300422 (PMC10647898; doi:10.1590/1677-5449.202300422)
Supplement: Translation

# Translation: Diretrizes brasileiras de diagnóstico e tratamento de lesões vasculares traumáticas

**DOI:** 10.1590/1677-5449.202300421

**Authors:** Adenauer Marinho de Oliveira Góes, José Gustavo Parreira, Gustavo Henrique Dumont Kleinsorge, Marcelo Bellini Dalio, Pedro Henrique Ferreira Alves, Francisco João Sahagoff de Deus Vieira Gomes, Walter Junior Boim de Araujo, Edwaldo Edner Joviliano, Julio Cesar Peclat de Oliveira

**Affiliations:** 1 Sociedade Brasileira de Angiologia e de Cirurgia Vascular - SBAC, São Paulo, SP, Brasil.; 2 Sociedade Brasileira de Atendimento Integrado ao Traumatizado - SBAIT, São Paulo, SP, Brasil.; 3 Centro Universitário do Pará - CESUPA, Faculdade de Medicina, Belém, PA, Brasil.; 4 Universidade Federal do Pará - UFPA, Faculdade de Medicina, Belém, PA, Brasil.; 5 Faculdade de Ciências Médicas da Santa Casa de São Paulo, Departamento de Cirurgia, São Paulo, SP, Brasil.; 6 Fundação Hospitalar do Estado de Minas Gerais - FHEMIG, Clínica de Cirurgia Vascular, Hospital João XXIII, Belo Horizonte, MG, Brasil.; 7 Universidade de São Paulo - USP, Divisão de Cirurgia Vascular e Endovascular, Hospital das Clínicas, Ribeirão Preto, SP, Brasil.; 8 Universidade de São Paulo - USP, Faculdade de Medicina, Hospital das Clínicas, III Clínica Cirúrgica, São Paulo, SP, Brasil.; 9 Universidade do Estado do Rio de Janeiro - UERJ, Faculdade de Ciências Médicas, Departamento de Cirurgia, Rio de Janeiro, RJ, Brasil.; 10 Polícia Militar do Estado do Rio de Janeiro - PMERJ, Rio de Janeiro, RJ, Brasil.; 11 Universidade Federal do Paraná - UFPR, Hospital das Clínicas, Divisão de Angiorradiologia e Cirurgia Endovascular, Curitiba, PR, Brasil.; 12 Universidade Federal do Estado do Rio de Janeiro - UNIRIO, Departamento de Cirurgia, Rio de Janeiro, RJ, Brasil.

**Keywords:** trauma, lesões do sistema vascular, vasos sanguíneos, diretrizes

## Abstract

Trauma é uma causa importante de morbimortalidade, que acomete principalmente jovens. A hemorragia incoercível é o principal mecanismo de óbito precoce nessas vítimas, e as lesões vasculares não compressíveis representam grandes desafios para os cirurgiões. O traumatismo vascular impacta diretamente a viabilidade de membros traumatizados, aumentando o risco de amputação. Nas últimas décadas, muitas condutas de diagnóstico e tratamento de lesões vasculares traumáticas foram modificadas. A angiotomografia suplantou a angiografia como padrão ouro para diagnóstico, as técnicas endovasculares foram incorporadas ao arsenal terapêutico e o conceito de “controle de danos” foi estabelecido. No entanto, há lacunas na literatura nacional sobre a normatização de condutas em trauma vascular, principalmente considerando as limitações do Brasil. Por isso, a Sociedade Brasileira de Angiologia e de Cirurgia Vascular e a Sociedade Brasileira de Atendimento Integrado ao Traumatizado revisaram a literatura disponível sobre trauma vascular e organizaram diretrizes sobre o diagnóstico e tratamento dessas lesões.

## INTRODUÇÃO

Traumatismos vasculares representam uma importante causa de morbimortalidade. Estudos atuais demonstram que a ocorrência dessas lesões tem aumentado progressivamente com o passar do tempo, com estimativa de que cerca de 6% das vítimas de trauma civis apresentem alguma lesão vascular^1,2,3^ .

Nas últimas décadas, o diagnóstico e o tratamento do trauma vascular passaram por mudanças significativas, como a preconização do uso de torniquetes no ambiente pré-hospitalar; a substituição da angiografia tradicional pela angiotomografia como exame de primeira escolha para a avaliação de lesões; a adoção de protocolos de controles de danos, incluindo *shunts* vasculares temporários; e a aplicação cada vez mais frequente de técnicas endovasculares para controle temporário ou tratamento definitivo de sangramento^1,2,4,5-9^ . Apesar de todos esses incrementos, a mortalidade dos pacientes traumatizados segue aumentando; atualmente, em pacientes com menos de 50 anos de idade, o trauma mata mais do que as doenças oncológicas e cardiovasculares, sobrecarregando o sistema de saúde, e as hemorragias associadas às lesões vasculares representam um desafio para os cirurgiões^3,5^ .

Em um centro de trauma nível 1 norte-americano, o número de pedidos de avaliação em caráter de urgência para a especialidade de cirurgia vascular aumentou em cerca de 500% nos últimos 15 anos, tendo os cirurgiões vasculares sido mais frequentemente acionados para lidar com membros isquêmicos, hemorragia ativa e exposições cirúrgicas complexas^3^ . Entre as razões para a crescente demanda por cirurgiões vasculares nos serviços de trauma, está o fato de o cirurgião geral/cirurgião de trauma ter cada vez menos treinamento em cirurgia vascular, repercutindo negativamente na sua capacidade de conduzir traumatismos de vasos sanguíneos^3^ .

O trauma vascular apresenta peculiaridades epidemiológicas que variam de acordo com o país. Na Europa, no Japão e na Austrália, predominam os mecanismos contusos, como quedas de altura e acidentes de trânsito; já nos Estados Unidos, na África e em países da América do Sul, predominam os ferimentos penetrantes^1^ . Dados brasileiros confirmam que os traumatismos por arma branca e arma de fogo são os mais associados a traumatismos vasculares no país, acometendo principalmente vasos das extremidades de homens jovens^10-12^ .

Em 2022, a diretoria da Sociedade Brasileira de Angiologia e Cirurgia Vascular (SBACV), através do Projeto Diretrizes, determinou a atualização de diretrizes sobre diversos temas; porém, não havia diretrizes anteriores que considerassem as peculiaridades brasileiras de apresentação e disponibilidade de recursos para a condução de casos de trauma vascular. Por entender que muitas lesões vasculares são tratadas por cirurgiões não especializados em cirurgia vascular, a Sociedade Brasileira de Atendimento Integrado ao Traumatizado (SBAIT) foi convidada a integrar o grupo de trabalho desta Diretriz.

O objetivo desta Diretriz foi padronizar a conduta relacionada a lesões traumáticas vasculares das diversas topografias, com ênfase no diagnóstico e tratamento, com base nos melhores níveis de evidência disponíveis e no consenso de um grupo de cirurgiões com experiência na condução de traumatismos vasculares.

## MÉTODO

Um grupo de trabalho foi composto por seis cirurgiões com experiência na condução de casos de traumatismos vasculares, os quais foram indicados pelas diretorias da SBACV e da SBAIT. Um dos membros atuou como coordenador do projeto.

Os participantes formularam questões sobre estratégias diagnósticas e terapêuticas para traumatismos vasculares das seguintes topografias: pescoço, tórax, abdome e membros. Em seguida, cada participante respondeu a essas questões com base na literatura publicada sobre traumatismos vasculares, por meio de buscas nas bases de dados MEDLINE, SciELO, LILACS, Scopus e Embase. Foi considerada a melhor evidência disponível. Os descritores pesquisados foram: femoral, poplítea, tibial, fibular, aorta, subclávia, axilar, braquial, radial, ulnar, cava, ilíaca, artéria, veia, trauma, lesão, diagnóstico e tratamento. Foram incluídos artigos publicados de 1990 a 2022 nos idiomas português e inglês.

O coordenador não respondeu às questões, pois ficou encarregado de receber as respostas de cada participante, os quais não tiveram acesso às respostas de seus pares. Com base nas respostas dos participantes, o coordenador formulou recomendações sobre diagnóstico e tratamento de lesões vasculares de cada topografia e, posteriormente, as enviou a cada participante em rodadas de trocas de e-mail. Cada recomendação foi pontuada de 1 a 5, de acordo com o grau de concordância. Quando a pontuação atribuída era igual ou menor que 3, era solicitado ao participante que fornecesse justificativas e propusesse modificações, indicando a referência da literatura que utilizou como embasamento. Utilizando o método Delphi^13^ , o coordenador realizou modificações até que cada recomendação recebesse uma nota mínima de 4, refletindo um elevado grau de concordância, entre 80% dos participantes.

Foram adotados como sinais maiores (*hard signs*) e sinais menores (*soft signs*) de trauma vascular aqueles expostos na Tabela 1. As classificações das lesões abdominais e dos membros estão dispostas nas Tabelas 2 e 3, respectivamente, de acordo com o preconizado pela *American Association for the Surgery of Trauma* ^1,2^ .

**Tabela 1:** Sinais clínicos de lesão vascular periférica.

**Sinais maiores**	**Sinais menores**
Sangramento pulsátil	Sangramento não pulsátil
Hematoma pulsátil ou em expansão	Hematoma não pulsátil ou não expansivo
Perda dos pulsos distais	Diminuição dos pulsos
Sopro e/ou frêmito	História de sangramento arterial (maciço)
Hipotensão	Torniquete aplicado anteriormente
	Déficit neurológico
	Ferimento no trajeto dos vasos tronculares

Adaptado de Kobayashi et al. ^2^

**Tabela 2:** Escala de graduação da AAST para lesões traumáticas vasculares abdominais.

**Grau**	**Lesão**
I	Ramos arteriais e/ou tributárias venosas mesentéricos(as) inominados(as)
	Artéria e/ou veia frênica
	Artéria e/ou veia lombar
	Artéria e/ou veia gonadal
	Artéria e/ou veia ovariana
	Outra pequena artéria e/ou veia inominada que requeira ligadura
II	Artéria hepática direita, esquerda ou comum
	Artéria ou veia esplênica
	Artéria gástrica direita ou esquerda
	Artéria gastroduodenal
	Artéria mesentérica inferior ou veia mesentérica inferior
	Ramos primários nomeados da artéria mesentérica superior ou tributárias da veia mesentérica superior (por exemplo: artéria ileocólica)
	Outros vasos abdominais que requeiram ligadura ou reparo
III	Tronco da veia mesentérica superior
	Artéria e/ou veia renal
	Artéria e/ou veia ilíaca
	Artéria e/ou veia hipogástrica
	Veia cava inferior infrarrenal
IV	Tronco da artéria mesentérica superior
	Tronco celíaco
	Veia cava inferior suprarrenal ou infra-hepática
	Aorta abdominal infrarrenal
V	Veia porta
	Veia hepática extraparenquimatosa
	Veia cava inferior retro-hepática ou supra-hepática
	Aorta abdominal suprarrenal subdiafragmática

AAST = American Association for the Surgery of Trauma. Adaptado de Kobayashi et al.^1^

**Tabela 3:** Escala de graduação da AAST para lesões traumáticas vasculares periféricas.

**Grau**	**Lesão**
I	Artéria/veia digital
	Artéria/veia palmar
	Artéria/veia palmar profunda
	Artéria dorsal do pé
	Artéria/veia plantar
	Ramos arteriais/tributárias venosos(as) inominados(as)
II	Veia basílica/cefálica
	Veia safena
	Artéria radial
	Artéria ulnar
III	Veia axilar
	Veia femoral superficial/profunda
	Veia poplítea
	Artéria braquial
	Artéria tibial anterior
	Artéria tibial posterior
	Artéria fibular
	Tronco tíbio-fibular
IV	Artéria femoral superficial/profunda
	Artéria poplítea
V	Artéria axilar
	Artéria femoral comum

AAST = American Association for the Surgery of Trauma. Adaptado de Kobayashi et al.^2^

Para as recomendações sobre traumatismos vasculares cervicais contusos, considerou-se a classificação de Denver (classificação de Biffl modificada), como expresso na Tabela 4 ^14^ . A classificação utilizada para traumatismos contusos da aorta torácica foi a proposta pela *Society for Vascular Surgery*, conforme a Tabela 5 ^15^ .

**Tabela 4:** Escala Denver de classificação para lesão contusa da artéria carótida.

Grau I	Imagem arteriográfica de irregularidade da parede do vaso ou dissecção/hematoma intramural com estenose luminal inferior a 25%.
Grau II	Trombo intraluminal ou flap intimal levantado é visualizado, ou dissecção/hematoma intramural com estreitamento luminal de 25% ou mais.
Grau III	Pseudoaneurisma.
Grau IV	Oclusão do vaso.
Grau V	Transecção com livre extravasamento.

Adaptado de Biffl et al.^14^

**Tabela 5:** Sistema de classificação das lesões traumáticas da aorta torácica.

**Grau**	**Lesão**
I	Laceração da íntima
II	Hematoma intramural
III	Pseudoaneurisma
IV	Ruptura

Adaptado de Lee et al.^15^

Os níveis de evidência foram classificados usando a adaptação do *Grading of Recommendations Assessment, Development and Evaluation System* ^16^ , previamente utilizada nas diretrizes internacionais de trauma vascular abdominal^1^ e de membros^2^ publicadas pela *American Association for the Surgery of Trauma* e pela *World Society of Emergency Surgery* (Tabela 6).

**Tabela 6:** Sistema GRADE modificado para grau de recomendação.

**Grau de Recomendação**	**Clareza de risco/benefício**	**Qualidade de evidências de suporte**	**Implicações para a prática clínica**
1A	Os benefícios superam claramente os riscos e encargos, ou vice-versa.	Evidências consistentes de ensaios randomizados bem realizados e controlados, ou evidências esmagadoras de alguma outra forma. É improvável que novas pesquisas mudem nossa confiança na estimativa de benefício e risco.	Recomendações fortes que podem ser aplicadas à maioria dos pacientes na maioria das circunstâncias, sem reservas. Os médicos devem seguir uma recomendação forte, a menos que haja uma lógica clara para uma abordagem alternativa.
Recomendação forte. Evidência de alta qualidade.			
1B	Os benefícios superam claramente os riscos e encargos, ou vice-versa.	Evidências de ensaios randomizados e controlados com limitações importantes (resultados inconsistentes, falhas de métodos, indiretas ou imprecisas), ou evidências muito fortes de algum outro desenho de pesquisa. Novas pesquisas podem impactar nossa confiança na estimativa de benefício e risco.	Recomendação forte que se aplica à maioria dos pacientes. Os médicos devem seguir uma forte recomendação, a menos que haja uma lógica clara e convincente para uma abordagem alternativa.
Recomendação forte. Evidência de qualidade moderada.			
1C	Os benefícios parecem superar os riscos e encargos, ou vice-versa.	Evidências de estudos observacionais, experiência clínica não sistemática ou de ensaios randomizados controlados com falhas graves. Qualquer estimativa de efeito é incerta.	Recomendação forte que se aplica à maioria dos pacientes. Parte da base de evidências que sustenta a recomendação é, no entanto, de baixa qualidade.
Recomendação forte. Evidência de baixa qualidade.			
2A	Benefícios intimamente equilibrados com os riscos e encargos.	Evidências consistentes de ensaios randomizados bem realizados e controlados, ou evidências esmagadoras de alguma outra forma. É improvável que novas pesquisas mudem nossa confiança na estimativa de benefício e risco.	Recomendação fraca. A melhor ação pode diferir dependendo das circunstâncias ou dos valores dos pacientes ou da sociedade.
Recomendação fraca. Evidência de alta qualidade.			
2B	Benefícios intimamente equilibrados com os riscos e encargos; alguns incertos nas estimativas de benefícios, riscos e encargos.	Evidências de ensaios randomizados e controlados com limitações importantes (resultados inconsistentes, falhas de métodos, indiretas ou imprecisas), ou fortes evidências de algum outro projeto de pesquisa. Novas pesquisas podem alterar a estimativa de benefício e risco.	Recomendação fraca. Abordagens alternativas provavelmente serão melhores para alguns pacientes em algumas circunstâncias.
Recomendação fraca. Evidência de moderada qualidade.			
2C	Incerteza nas estimativas de benefícios, riscos e encargos; os benefícios podem ser intimamente equilibrados com os riscos e encargos.	Evidências de estudos observacionais, experiência clínica não sistemática ou de ensaios randomizados controlados com falhas graves. Qualquer estimativa de efeito é incerta.	Recomendação fraca. outras alternativas podem ser razoáveis.
Recomendação fraca. Evidência de baixa qualidade.			

Adaptado de Kobayashi et al.^1^

O texto final foi revisado pelo grupo e considerado um consenso estruturado.

## QUESTÕES E RECOMENDAÇÕES

### Recomendações gerais sobre lesões vasculares traumáticas

#### Quando necessário, qual o substituto vascular de escolha?

Recomendações:

- Para a maioria dos vasos cervicais e dos membros, o substituto de escolha é uma veia autóloga, preferencialmente de uma extremidade não traumatizada. A mais utilizada é a veia safena magna (1C).
- Veias autólogas são preferíveis para a reconstrução de vasos com diâmetro de até 7 mm. Para vasos mais calibrosos, como os grandes vasos torácicos e abdominais, próteses vasculares são a solução mais utilizada (1C).
- Se não houver veia autóloga disponível (calibre não compatível ou impossibilidade de prolongar o tempo cirúrgico para retirada do enxerto), próteses de politetrafluoroetileno (PTFE) ou Dacron podem ser utilizadas. O PTFE está associado a menor incidência de complicações infecciosas (1C).
- Quando houver contaminação da cavidade abdominal, se possível, o uso de próteses deve ser evitado (2C).
- Embora lesões extensas das veias ilíacas e cava inferior sejam frequentemente tratadas com ligadura, o uso da veia safena magna “espiralada” como enxerto já foi descrito em casos selecionados (1C)^1,2,17,18,19,20^ .

#### Quais as indicações para controle de danos (CD) em trauma vascular e como realizá-lo?

Recomendações:

- As indicações para CD incluem:
  - Pacientes em coagulopatia, acidose metabólica (base excesso ≤ 10 ou pH < 7,1) e hipotermia (tríade letal), com necessidade de reconstrução vascular (1C).
  - Lesões concomitantes que requeiram tratamento imediato (1C).
  - Pacientes *in extremis* (1B).
  - Necessidade de múltiplas transfusões de hemoderivados (1B).
  - Indisponibilidade dos recursos necessários para o tratamento definitivo da lesão vascular (1B).
- As técnicas mais frequentemente empregadas para CD em trauma vascular são:
  - Ligadura de vasos não essenciais (2C).
  - Implante de *shunt* vascular temporário em vasos cujo fluxo precise ser preservado (2C).
  - Hemostasia temporária por tamponamento com balões ou compressas (2C).
  - Técnicas de CD para lesões não vasculares associadas, como peritoniostomia nos traumatismos abdominais e síntese abreviada de planos anatômicos em outras topografias^1,2,5,17,21^ .

#### Há indicação de anticoagulação (AC) e/ou antiagregação plaquetária (AAP) peroperatória em lesões vasculares traumáticas? Se sim, como realizá-las?

Recomendações:

- Nos traumas cervicais contusos, as lesões de graus I e II das artérias carótidas e vertebrais podem ser conduzidas exclusivamente com AAP e/ou AC (1C). Não foram localizadas evidências que favoreçam um determinado esquema de AAP ou AC a longo prazo; entretanto, recomenda-se inicialmente AC com heparina (preferencialmente não fracionada), pela possibilidade de reversão em caso de complicações hemorrágicas (2C). Não foram localizados dados que embasem o uso de AC ou AAP após reconstruções carotídeas.
- Não há evidências para recomendação rotineira de AC e/ou AAP após o tratamento cirúrgico de lesões vasculares torácicas; entretanto, ambas podem ser utilizadas na condução não operatória de lesões menores das artérias subclávias e inominada (2C). Não foram encontradas recomendações específicas sobre medicamentos, tempo de uso e dosagens para essa situação.
- Nos traumatismos abdominais, caso o reparo vascular promova estenose venosa significativa, recomenda-se a ligadura venosa. Quando a veia em questão não puder ser ligada (por exemplo, cava inferior cranialmente às veias renais), a AC deve ser considerada e, nesses casos, não há consenso quanto a doses e duração. Pacientes com lesões venosas abdominais/pélvicas devem ser monitorados pelo risco de desenvolverem tromboembolismo venoso (TEV) (2C).
- Não há evidências que embasem o uso de AC sistêmica intraoperatória ou AAP/AC pós-operatória na maioria dos reparos vasculares em membros. A exceção é o tempo isquêmico prolongado com oclusão de vasos de menor calibre (2C).
- Todo paciente traumatizado acamado deve receber heparina em doses profiláticas para TEV, respeitando-se as contraindicações da administração de AC (2C)^1,2,16,22,23,24,25,26^ .

### Recomendações sobre traumatismo vascular cervical

#### Quando utilizar exames de imagem para o diagnóstico de lesão vascular cervical?

Recomendações:

- Pacientes vítimas de traumatismo cervical penetrante que não apresentem sinais maiores de trauma vascular e pacientes com traumatismo contuso com mecanismo compatível* com trauma vascular cervical (1C).
  - Critérios de Denver modificados: fraturas de coluna cervical/face/base de crânio, lesão axonal difusa com pontuação na escala de coma de Glasgow < 6 e mecanismo de trauma sugestivo (impacto direto cervical, estiramento + rotação) (2C)^23,24,26,27^ .

#### Quais exames de imagem devem ser realizados?

Recomendações:

- A angiotomografia é adequada para avaliar lesões nas zonas cervicais 1, 2 e 3. Lesões de zona 2 também podem ser avaliadas pelo ultrassom (1C) ^8,17,19^ .

#### Qual o papel do tratamento endovascular no traumatismo vascular cervical?

Recomendações:

- A cervicotomia continua sendo a opção preferencial para lesões com acesso cirúrgico menos complexo, como as lesões carotídeas em zona cervical 2 (1C).
- As principais indicações para tratamento endovascular são: (a) no traumatismo carotídeo contuso ou penetrante em áreas de difícil exposição cirúrgica (zonas 1 e 3); e (b) no traumatismo penetrante da artéria vertebral (embolização da artéria não dominante e implante de stent revestido na artéria dominante) (1C).
- Dispositivos destinados a oclusão vascular (embolização) ou reconstrução vascular (stents revestidos) podem ser utilizados em diferentes padrões de lesões (pseudoaneurismas, fístulas arteriovenosas e secções parciais/totais do vaso), resultantes tanto de mecanismo contuso quanto penetrante (1C) ^28,29,30^ .

#### Quais lesões vasculares cervicais podem ser acompanhadas de forma expectante?

Recomendações:

- Traumatismo contuso: pacientes sem sintomas neurológicos que apresentem lesões carotídeas de grau I (lesão intimal com menos de 25% de redução luminal), II (dissecção ou hematoma com mais de 25% de redução luminal) ou IV (oclusão); e lesões das artérias vertebrais (1C).
- Traumatismo penetrante: pacientes sem sintomas neurológicos que apresentem oclusão das artérias carótidas ou vertebrais; achado de lesões vasculares mínimas em exame de imagem (*flap* intimal não obstrutivo ou pseudoaneurisma com diâmetro menor que 5 mm) (1C); e lesões venosas sem sangramento ativo (2C), desde que não haja indicação concomitante de cervicotomia por lesões associadas^8,24,25^ .

#### Quais lesões vasculares cervicais podem ser tratadas por ligadura?

Recomendações:

- Todas as lesões venosas, exceto lesões bilaterais das jugulares internas; a ligadura bilateral está associada a hipertensão intracraniana, recomendando-se reconstrução de ao menos uma jugular interna (1C).
- Lesões das artérias carótidas externas e seus ramos e das artérias vertebrais podem ser tratadas por ligadura (1C).
- Lesões da carótida interna em zona 3, quando as limitações do campo cirúrgico impedirem o implante de *shunt* temporário ou a reconstrução vascular (2C).
- Lesões carotídeas em pacientes *in extremis,* quando a instalação de *shunt* vascular temporário não for possível (2C)^28,31,32^ .

#### Há indicação do uso de *shunt* durante a correção das lesões carotídeas?

Recomendações:

- Não há evidências de que o uso de *shunt* em reconstruções mais complexas (como interposição de enxertos venosos) esteja associado a diferença significativa quanto à evolução neurológica (2C).
- Recomenda-se especialmente o uso de *shunts* em caso de fluxo retrógrado (refluxo) inadequado ou quando a reconstrução carotídea não puder ser concluída no primeiro ato operatório (CD) (2C) ^33,34^ .

#### Quando a artéria carótida estiver trombosada após um traumatismo penetrante, ela deve ser revascularizada?

Recomendações:

- Se o paciente já apresenta pontuação na escala de coma de Glasgow < 8, a probabilidade de a reconstrução carotídea ser benéfica é mínima, podendo ser realizada ligadura (2C).
- Se a pontuação na escala de coma de Glasgow for > 8, deve-se tentar reconstruir a artéria carótida, salvo contraindicações para reparo em lesões penetrantes: lesões inacessíveis, coma há mais de 4 h, grandes áreas de infarto cerebral demonstradas por exames de imagem na admissão ou ausência de refluxo no segmento arterial distal após trombectomia (2C)^17,18,32^ .
- Observação: para orientações quanto à condução de trombose carotídea após traumatismos contusos, verificar a recomendação 1.3 destas Diretrizes.

### Recomendações sobre traumatismo vascular torácico

#### Quais são as indicações de toracotomia sem investigação com exames de imagem?

Recomendações:

- Vítimas de traumatismo torácico que apresentem instabilidade hemodinâmica persistente após as medidas de atendimento inicial ao traumatizado (inclusive tratamento de pneumotórax hipertensivo) ou hemorragia importante por dreno pleural (> 1.200 mL ou drenagem persistente 200 mL/h) (1C)^17,21,35, 36, 37^ .

#### Quando utilizar exames de imagem para o diagnóstico de lesão vascular torácica?

Recomendações:

- Pacientes sintomáticos devem ser submetidos a investigação ativa desde que a condição hemodinâmica permita (1C).
- Pacientes assintomáticos devem ser investigados sempre que o mecanismo do trauma for compatível com lesão vascular, por exemplo:
  - Ferimentos penetrantes em trajeto vascular ou ferimentos transfixantes do mediastino.
  - Trauma fechado com mecanismo sugestivo - por exemplo, desaceleração significativa em quedas de altura e acidentes de trânsito e fraturas de escápula, clavícula e primeiro arco costal (1C).
  - Politraumatizados com pontuação na escala de coma de Glasgow < 12 ou traumatismo cranioencefálico grave (1C)^17,35,36,38,39^ .

#### Quais exames de imagem devem ser realizados?

Recomendações:

- Pacientes instáveis podem ser submetidos a radiografia de tórax na sala de emergência e a *extended focused assessment with sonography in trauma* (e-FAST) (1C).
- Para pacientes estáveis, a angiotomografia de tórax é o exame de escolha (1C).
- Quando permanecerem dúvidas, a angiografia pode ser realizada. No entanto, esse método é atualmente mais empregado como parte de procedimentos endovasculares terapêuticos (1C)^17,35,36,40,41^ .

#### Quais lesões vasculares torácicas podem ser acompanhadas de forma expectante?

Recomendações:

- Lesões mínimas da artéria subclávia (pequenas dissecções/pseudoaneurismas) podem ser acompanhadas de forma não operatória, assim como traumatismos contusos que provoquem oclusões da artéria subclávia, desde que a perfusão do membro esteja compensada. Nesses casos, recomenda-se acompanhamento clínico e radiológico (2C).
- Lesões contusas mínimas da aorta de graus I e II podem ser passíveis de tratamento não operatório; betabloqueadores e controle pressórico podem ser usados em pacientes hemodinamicamente estáveis (1C) ^17,35,38,37,42,41-44^ .

#### Qual o papel do tratamento endovascular no traumatismo vascular torácico?

Recomendações:

- O controle proximal de várias lesões pode ser obtido pela insuflação de cateteres de balão inseridos por técnicas endovasculares (*endoclamp*), reduzindo o sangramento e facilitando a dissecção do vaso traumatizado (1C).
- O tratamento endovascular é preferível para traumatismos contusos da aorta de graus III (pseudoaneurisma) e IV (rotura) (1C).
- O tratamento endovascular pode ser empregado como primeira escolha em traumatismos contusos e penetrantes das artérias subclávia e axilar, mesmo em pacientes instáveis, desde que os recursos necessários estejam disponíveis e não haja outra indicação prioritária de toracotomia (1C). Embora infrequente, há relatos de tratamento de traumatismos de artéria inominada (2C). Além de recursos materiais e logísticos, é necessária a experiência da equipe no tratamento endovascular dessas lesões^35,37,38,42,43,45^ .

#### Quais vasos podem ser ligados como tratamento definitivo?

Recomendações:

- Veias: quase todas as veias torácicas podem ser ligadas sem maiores consequências. As exceções são a cava superior e o segmento intrapericárdico da cava inferior. A jugular interna pode ser ligada, mas ligaduras bilaterais devem ser evitadas (2C).
- Artérias: as artérias subclávias podem ser ligadas proximalmente à origem da artéria vertebral; nessas condições, a perfusão do membro superior pode ser mantida por meio do fenômeno de “roubo da artéria subclávia” (1C). Na condição clínica de hemotórax maciço e toracotomia de emergência, a ligadura da artéria pulmonar requer pneumonectomia (2C) ^17,21,35,44^ .

#### Quais vasos devem ser reconstruídos sempre que possível?

Recomendações:

- Veias: cava superior, segmento intrapericárdico da cava inferior e ao menos uma veia jugular interna (no caso de lesões bilaterais) (1C).
- Artérias: ligadura de artérias torácicas é uma situação de exceção; aorta, inominada e segmentos intratorácicos das artérias carótidas comuns devem ter o seu fluxo mantido sempre que possível (1C) ^17,21,46^ .

#### Quais as vias de acesso cirúrgico para abordagem das diferentes lesões vasculares torácicas?

Recomendações:

- Em pacientes estáveis, exames de imagem podem auxiliar no planejamento. As vias de acesso mais frequentes e as respectivas estruturas abordadas são (1C):
  - Estenotomia mediana: coração, aorta ascendente, arco aórtico, artérias pulmonares, troncos braquiocefálicos arterial e venosos, cava superior e segmento intrapericárdico da cava inferior.
  - Esternotomia mediana com extensão cervical ou supraclavicular: tronco braquiocefálico arterial e artérias e veias subclávias proximais. Toracotomia anterolateral esquerda alta (segundo/terceiro espaço intercostal) para acesso ao segmento proximal da artéria subclávia esquerda também já foi descrita.
  - Toracotomia posterolateral esquerda no quarto/quinto espaço intercostal: aorta torácica descendente, hilo pulmonar esquerdo e veias pulmonares.
  - Toracotomia anterolateral esquerda no quarto/quinto espaço intercostal: hilo pulmonar esquerdo e coração.
  - Toracotomia anterolateral direita no quarto/quinto espaço intercostal: hilo pulmonar direito.
  - Supraclavicular e/ou infraclavicular (com ou sem cleidectomia): artérias e veias subclávias e axilares.
- Em pacientes instáveis, não há tempo hábil para exames de imagem, e a via de acesso deve ser indicada com base na apresentação clínica:
  - Toracotomia anterolateral esquerda no quarto/quinto espaço intercostal: toracotomia de reanimação.
  - No caso de traumatismos acometendo ambos os lados do tórax: a exploração pode ser iniciada por uma toracotomia anterolateral no quarto/quinto espaço intercostal no lado cujo débito do dreno torácico revelar o hemotórax mais volumoso.
  - A bitoracotomia no quarto/quinto espaço intercostal (*clam shell*) é a única via que promove acesso simultâneo ao mediastino e às duas cavidades pleuropulmonares e pode ser utilizada em pacientes instáveis quando não se conhece a topografia da lesão^17,21,37,47^ .

### Recomendações sobre traumatismo vascular abdominal

#### Quais são as indicações de laparotomia sem investigação com exames de imagem?

Recomendações:

- Pacientes hemodinamicamente instáveis com suspeita de lesão vascular abdominal devem ser operados sem investigação com exames de imagem (1B). A instabilidade hemodinâmica é secundária ao trauma abdominal penetrante ou trauma contuso com hemoperitônio detectado pelo FAST ou lavado peritoneal. Essas indicações dizem respeito à possibilidade de lesões vasculares intra-abdominais e não excluem outras indicações de laparotomia, como sinais clínicos de peritonite e evisceração, por exemplo^1,22,48^ .

#### Quando utilizar exames de imagem para o diagnóstico de lesão vascular? Quais exames solicitar?

Recomendações:

- Pacientes hemodinamicamente estáveis, com sinais de lesão intra-abdominal/mecanismo compatível com lesões vasculares*, porém sem indicação de laparotomia imediata, devem ser submetidos a angiotomografia para triagem de lesões vasculares e planejamento de acesso (1A). Os mecanismos de trauma compatíveis com lesões vasculares incluem fraturas de pelve (principalmente instáveis), fraturas de coluna lombar, mecanismo de desaceleração importante e sinal do cinto de segurança.
- A angiotomografia é o exame recomendado para o diagnóstico de lesões vasculares abdominais (1A).
- O ultrassom deve ser utilizado como método de triagem para detectar a presença de líquido intra-abdominal, mas não para o diagnóstico de lesões vasculares abdominais, pois possui baixa acurácia para essa finalidade específica (1B).
  Observação: no contexto atual, a angiografia é usada principalmente como parte integrante dos procedimentos endovasculares terapêuticos^1,49,50,51^ .

#### Quais lesões podem ser de tratamento não operatório?

Recomendações:

- O tratamento não operatório pode ser considerado em pacientes estáveis, com lesões isoladas de graus 1 a 3 (AAST), sem a presença de extravasamento de contraste à angiotomografia (2C).
  Observação 1: na literatura estão também apresentados casos de traumatismos contusos e penetrantes da veia cava inferior (1C), aorta abdominal (C2), veia porta (1C), artéria mesentérica superior e tronco celíaco conduzidos de forma não operatória.
  Observação 2: antes de considerar adotar o tratamento não operatório, é fundamental avaliar a estabilidade hemodinâmica e a possibilidade de lesões não vasculares associadas.
  Observação 3: para a adoção de tratamento não operatório em lesões abdominais, recomenda-se fortemente a criação de protocolos institucionais^1,5,10,22,52,53,54,55^ .

#### Qual o papel do tratamento endovascular no traumatismo vascular abdominal?

Recomendações:

- O tratamento endovascular pode ser uma opção de tratamento definitivo para pacientes estáveis com lesões isoladas de graus 1 a 4 (AAST) (2C).
- A oclusão endovascular da aorta por meio da insuflação de balões (REBOA) pode ser usada como parte das manobras de reanimação até que o tratamento definitivo possa ser realizado (2C).
  Observação: na literatura está também descrita a possibilidade do emprego de técnicas endovasculares para as seguintes lesões/situações: lesões da artéria mesentérica superior (2C), trombose da artéria renal por traumatismo contuso (2C), embolização da artéria ilíaca interna e seus ramos no contexto das fraturas pélvicas (2C) e traumatismos fechados e penetrantes da veia cava inferior e da aorta abdominal (2C). Também é descrita a oclusão endovascular com balões para o controle de sangramento associado a procedimentos cirúrgicos abertos (*endoclamp*) para traumatismos de segmentos de difícil acesso com os segmentos retro-hepático e supra-hepático da veia cava inferior (2C)^1,5,17,48,53,56^ .

#### Durante uma laparotomia, quando explorar hematomas de retroperitônio? Quais manobras de exposição/controle vascular são recomendadas?

Recomendações:

- Hematomas retroperitoneais associados a traumatismos penetrantes, independentemente da zona acometida, devem ser explorados (1B). Uma exceção a essa regra pode ser feita no caso de lesões penetrantes da cava retro-hepática, desde que na presença de hematomas contidos e pacientes estáveis. Está descrita, inclusive, para casos selecionados, a condução não operatória.
- Hematomas retroperitoneais em zona 1 associados a traumatismos contusos devem ser explorados (1B).
- Hematomas em zona 2 associados a traumatismos contusos devem ser explorados caso apresentem sinas de expansão ou pulsatilidade (1B).
- Hematomas estáveis em zona 3 associados a traumatismos contusos não devem ser explorados (1B). Uma exceção a essa regra é feita nos casos de ausência de pulso femoral, quando a avaliação radiológica/exploração cirúrgica das artérias ilíacas comum e externa deve ser feita para descartar lesão. Durante a exploração cirúrgica, deve-se evitar avançar medialmente, diminuindo o risco de sangramentos associados ao destamponamento do hematoma pélvico.

Manobras de exposição/controle vascular relacionadas à exploração dos hematomas retroperitoneais (1B):

- Clampeamento supracelíaco da aorta na retrocavidade dos epíploons, ao nível do hiato diafragmático: está indicado para controle proximal da aorta na crura diafragmática esquerda diante de grandes hematomas retroperitoneais.
- Manobra de Mattox (rotação visceral medial pela abertura da goteira parietocólica esquerda): para exposição da zona 2 esquerda e do segmento infrarrenal da aorta e dos vasos ilíacos esquerdos, pode ser realizada a manobra de Mattox modificada, que não inclui a mobilização do rim esquerdo. A manobra clássica, que inclui o rim na rotação visceral, permite a exposição de toda a extensão da aorta abdominal e a origem de seus ramos viscerais.
- Manobra de Cattell-Braasch (rotação visceral medial pela abertura da goteira parietocólica direita): permite a exposição dos vasos ilíacos direitos e da porção infrarrenal da cava inferior. Pode ser complementada com a manobra de Kocher, que, através da mobilização duodenopancreática, termina de expor a cava no seu segmento perirrenal até a borda hepática inferior.
- Acesso anterior transperitoneal: a abertura do retroperitônio longitudinalmente entre o duodeno e a veia mesentérica inferior permite o acesso à porção infrarrenal da cava e da aorta.
- Manobra de Pringle: consiste no clampeamento da tríade portal. Embora não seja uma manobra de exposição retroperitoneal, no contexto das lesões vasculares, é frequentemente utilizada como parte das manobras necessárias para abordagem das lesões de cava retro-hepática.
- Para exposição da veia porta e veia mesentérica superior retropancreática em pacientes com sangramento ativo importante, pode ser necessária a secção do pâncreas (geralmente já lesado). Caso isso seja necessário, deve-se realizar pancreatectomia corpo-caudal com esplenectomia ^1,17,48^ .

#### Quais vasos podem ser ligados como tratamento definitivo?

Recomendações:

- Veias:
- As veias ilíacas comum, externa e interna, mesentérica inferior e esplênica (caso a veia esplênica seja ligada, deve-se realizar esplenectomia) podem ser ligadas como tratamento definitivo (1C).
- Em pacientes em situações de CD, a veia cava infrarrenal, a veia mesentérica superior e a veia porta podem ser ligadas (1C). Caso a veia mesentérica superior ou a porta seja ligada, indica-se peritoniostomia e atenção especial quanto à reposição volêmica para compensar o volume represado no sistema esplâncnico. A ligadura da veia porta somente deve ser realizada caso a artéria hepática esteja íntegra. A perviedade de ao menos uma dessas estruturas é importante para a manutenção da perfusão hepática (1C).
- Veias renais: para manutenção da drenagem venosa, a veia renal esquerda deve ser ligada preferencialmente próximo à desembocadura na veia cava inferior. Caso a veia renal direita seja ligada, deve-se realizar nefrectomia direita (1C).
- Artérias:
- Tronco celíaco e seus ramos: considerar realizar colecistectomia, pois há relatos de necrose da vesícula biliar após ligadura da artéria hepática (essa possibilidade pode ser maior caso a ligadura seja distal à origem da artéria gastroduodenal). Caso a ligadura da artéria esplênica seja proximal, a esplenectomia pode não ser necessária (1C).
- Artéria mesentérica superior: ligadura distal à origem da artéria cólica média (zonas de Fullen III e IV). Provavelmente será necessário realizar enterectomia segmentar para tratar a isquemia resultante (1C).
- Artérias renais: complementar com nefrectomia.
- A artéria mesentérica inferior e as artérias ilíacas internas podem ser ligadas (1C)^1,17,48,52,54,57,58,59^ .

#### Quais vasos devem ser obrigatoriamente reconstruídos?

Recomendações:

- Os seguintes vasos devem ter seu fluxo preservado sempre que possível, quando a reconstrução não for possível. No contexto do CD, sugere-se o implante de *shunt* vascular temporário:
  - Segmento suprarrenal da cava inferior (1C).
  - Aorta e artérias ilíacas comuns e externas (1C).
  - Segmentos proximais da artéria mesentérica superior (Fullen 1 e 2) (1C).
  - A perviedade da veia porta ou da artéria hepática deve ser mantida para assegurar a perfusão hepática (1C)^1,17,48,52,54,57,58,59^ .

#### Como proceder no caso de lesões associadas a grande contaminação ou risco de fístula digestiva?

Recomendações:

- Vasos passíveis de ligadura devem ser ligados.
- Se um enxerto for necessário, veias autólogas são preferíveis. Próteses podem ser usadas para reconstrução de vasos calibrosos. Se possível, as próteses devem ser mantidas “protegidas” do contato com as vísceras, preferencialmente pela interposição do retroperitônio (1C).
- Ligadura vascular associada a reconstrução com derivação extra-anatômica em região com menor risco de infecção também pode ser realizada (1C) ^1, 17^ .

#### Quando considerar a realização de fasciotomia de membros inferiores em casos de lesões vasculares abdominais?

Recomendações:

- Lesões de aorta e artérias ilíacas comuns ou externas que determinem tempo prolongado de isquemia dos membros inferiores podem resultar em síndrome compartimental após a reperfusão dos membros inferiores (1C).
- A ligadura da veia cava inferior pode provocar edema importante de membros inferiores e causar síndrome compartimental (1C).

Em ambas as situações, deve-se acompanhar a evolução dos membros inferiores e, se necessário, realizar a fasciotomia. A realização profilática não é obrigatória^1,17,55^ .

#### Qual deve ser a sequência de tratamento para hematomas associados a fraturas de bacia?

Recomendações:

- Fraturas instáveis da bacia devem ser identificadas ao exame físico durante a avaliação primária do traumatizado. É importante avaliar a simetria dos pulsos femorais, que podem estar ausentes na presença de lesões nas artérias ilíacas comum e externa (1C).
- Pacientes hemodinamicamente estáveis devem ser submetidos a angiotomografia (1C).
- A primeira medida específica nos pacientes hemodinamicamente instáveis é reduzir o volume pélvico por meio de aplicação de cintas específicas ou materiais improvisados, como lençóis (1C).
- Se disponível, a oclusão endovascular da aorta por balões (REBOA) pode ser aplicada na zona de insuflação III (1C).
- Se uma hemorragia intraperitoneal for descartada (por FAST, lavado peritoneal ou laparotomia), a próxima prioridade é diminuir o sangramento proveniente do retroperitônio pélvico. As três técnicas mais utilizadas para essa finalidade são tamponamento pré-peritoneal com compressas, fixação externa da fratura e angioembolização (1C).
- O tamponamento pré-peritoneal é eficaz na redução de hemorragias associadas a lesões de plexos venosos, ramos arteriais ou focos de fratura. Em fraturas instáveis, a fixação externa deve ser obtida sempre que possível. Quando essas duas técnicas são usadas de forma combinada, a angioembolização é necessária apenas na minoria dos casos (1B).
- A angiografia deve ser considerada em pacientes com fraturas de pelve quando houver (1C):
  - Instabilidade hemodinâmica ou evidência de sangramento ativo após exclusão de outros focos hemorrágicos (pode ser repetida caso o paciente já tenha sido submetido a angiografia previamente).
  - Extravasamento de contraste arterial na tomografia, independentemente da condição hemodinâmica.
  - Idade superior a 60 anos, independentemente dos achados na angiotomografia ou do estado hemodinâmico.
  - Hematomas com volume estimado superior a 500 mL.
- A melhor sequência ou combinação ideal de aplicação do tamponamento pré-peritoneal, da fixação externa e da angioembolização ainda não está bem definida. No entanto, julgamos pertinentes os seguintes comentários:
  - Cada instituição deve desenvolver protocolos multidisciplinares (cirurgia do trauma/cirurgia vascular/ortopedia e traumatologia) que sejam adequados à sua realidade para o tratamento desses casos.
  - Pacientes hemodinamicamente instáveis devem ser preferencialmente reanimados em centro cirúrgico para controle dos focos hemorrágicos. Recomenda-se o emprego liberal do tamponamento pré-peritoneal, que pode, inclusive, preceder a fixação da fratura. Idealmente, a redução do volume pélvico com o uso de lençóis ou outros dispositivos específicos deve ser mantida durante a realização do tamponamento pré-peritoneal.
  - A fixação precoce da fratura deve ser considerada pela equipe de ortopedia. Normalmente, está indicada em fraturas tipo livro aberto e outras com grande abertura do anel pélvico. Entretanto, essa medida não se aplica a todas as fraturas instáveis.
  - O tamponamento pré-peritoneal deve ser considerado em pacientes com fraturas pélvicas instáveis, especialmente nos que persistem com instabilidade hemodinâmica ou sinais de sangramento ativo após imobilização pélvica/fixação externa ou nos que não são candidatos a fixação externa por características específicas da fratura. Essa é uma opção importante nos serviços que não dispõem de angiografia para embolização.
  - A angioembolização deve ser considerada nos pacientes com suspeita de sangramento arterial, frequentemente associado a instabilidade hemodinâmica e acidose metabólica já na admissão, grandes hematomas retroperitoneais e sinais de extravasamento de contraste na angiotomografia. No entanto, em pacientes instáveis, a fixação externa e o tamponamento pré-peritoneal não devem ser postergados para realizar a angioembolização, que é mais efetiva quando o sangramento é de origem arterial.
  - A angiografia também pode ser feita nos pacientes que permanecem instáveis após a fixação da fratura. Se a instabilidade hemodinâmica persistir e não houver recursos disponíveis para procedimentos endovasculares, a embolização pode ser feita por exposição cirúrgica das artérias ilíacas internas e punção arterial direta para injeção de coágulo autólogo, pasta de Gelfoam® ou outros materiais apropriados. As artérias ilíacas internas não devem ser submetidas a ligaduras cirúrgicas ^1,51,56,60^ .

### Recomendações sobre traumatismos vasculares dos membros

#### Quando utilizar exames de imagem para o diagnóstico de lesão vascular?

Recomendações:

- As indicações mais frequentes são:
  - Pacientes hemodinamicamente estáveis com *soft signs* e/ou índice tornozelo-braço (ITB) < 0,9 (1B).
  - Nos casos em que o tratamento endovascular está sendo considerado (1B).
- Desde que a estabilidade hemodinâmica permita, recomendam-se exames de imagem nas seguintes situações:
  - Traumatismos em níveis diferentes em uma mesma extremidade (mesmo com sinais maiores), a fim de esclarecer qual provocou a lesão vascular e se há mais de uma lesão vascular concomitante (2B).
  - Luxação de joelho, mesmo sem sinais de trauma vascular (2B) ^2,61^ .

#### Quais exames de imagem devem ser realizados?

Recomendações:

- A angiotomografia é o padrão ouro para a investigação de traumatismo vascular dos membros (1B).
- A angiografia por cateter é indicada em caso de suspeita de vasoespasmo, quando a angiotomografia não estiver disponível ou fornecer imagens prejudicadas por artefatos de objetos metálicos retidos (como projéteis de arma de fogo) ou como parte integrante do tratamento endovascular (1B).
- O ultrassom Doppler pode ser utilizado, inclusive com o protocolo *fast* Doppler, porém com limitações (1B).
- A antirressonância é pouco utilizada. É contraindicada na presença (ou suspeita) de fragmentos metálicos retidos (1B)^2,7,62,63,64,65,66,67,68,69^ .

#### É seguro dar alta com base apenas no exame físico (incluindo ITB)?

Recomendações:

- Em traumatismos penetrantes, um ITB ou índice de pressão arterial ≥ 0,9 na ausência de sinais de lesão vascular é suficiente para alta hospitalar sem investigação com exames de imagem. Recomenda-se acompanhamento ambulatorial pela possibilidade de manifestações tardias de lesões menores (1B).
- Nos mecanismos de trauma contusos de alta energia, como as luxações de joelho, o exame físico tem menor sensibilidade e, portanto, recomenda-se avaliação com exames de imagem (1B) ^2,61^ .

#### Quando indicar tratamento não operatório?

Recomendações:

- Lesões arteriais na mão e antebraço (AAST I e II) em pacientes hemodinamicamente estáveis, sem sangramento ativo e com perfusão distal compensada (1C).
- Lesões isoladas de artérias tibiais/fibulares quando houver uma artéria troncular intacta até o pé, sem sangramento ativo ou isquemia distal (1C).
- Lesões AAST de grau III quando houver outra artéria intacta no membro, sem sangramento ativo e com perfusão distal compensada (1C).
- Critérios de Franz (2011): lesão < 5 mm, *flap* intimal aderido, circulação distal intacta e ausência de hemorragia (1C) ^2,64,67,70^ .

#### As lesões venosas dos membros podem ser ligadas ou devem ser reconstruídas?

Recomendações:

- Como regra geral, quando for possível realizar o reparo necessário com venorrafia, veias calibrosas (subclávia, axilar, femorais comum e superficial e poplítea) devem ser reconstruídas, o que tende a oferecer melhor prognóstico funcional do membro. Quando as lesões exigirem reconstruções mais complexas, especialmente no contexto de controle de danos, qualquer veia dos membros pode ser ligada (1C)^2,70,71^ .

#### As lesões nervosas e tendinosas associadas devem ser reconstruídas no primeiro tempo cirúrgico ou em um procedimento posterior?

Recomendações:

- Ambas as condutas são possíveis. Se houver estabilidade hemodinâmica e o traumatismo vascular já estiver resolvido, lesões nervosas e tendinosas podem ser tratadas no mesmo tempo cirúrgico. A depender da condição sistêmica dos pacientes, de características específicas das lesões e da disponibilidade de recursos, o tratamento dessas lesões pode ser postergado (1C)^2^ .

#### Quais critérios usar para indicação de fasciotomias?

Recomendações:

- Mecanismo do trauma: traumatismo de extremidade com tempo de isquemia superior a 4-6 h e trauma de alta energia no antebraço ou na perna (1C).
- Apresentação clínica: dor na topografia do compartimento muscular acometido, edema tenso no compartimento, paresia, parestesia e ausência de pulsos distais (1C).
- Pressão intracompartimental > 30 mmHg (1C).
- Achados cirúrgicos/atos operatórios realizados: ligaduras de veias calibrosas, reconstruções arteriais após mais de 6 h de isquemia, associação de lesões arteriais e venosas (especialmente se foi realizada ligadura venosa) e lesões vasculares associadas a fraturas (1C)^2, 70^ .
- Observações:
  - A revisão da literatura aponta que a realização precoce de fasciotomia diminui a incidência de amputação e complicações infecciosas nos membros traumatizados.
  - O julgamento do cirurgião se sobrepõe a medidas de pressões compartimentais e exames complementares.
  - Os comentários acima não excluem outras indicações não relacionadas diretamente a traumatismos vasculares, como queimaduras ou esmagamentos de membros.

#### Quais lesões arteriais podem ser tratadas com ligadura?

Recomendações:

- Artérias do antebraço ou da perna, na presença de colaterais que mantenham a perfusão compensada (1C).
- As artérias subclávia e axilar podem ser ligadas nos pacientes *in extremis*. No caso das lesões das artérias subclávias, quando proximais à origem da artéria vertebral, há maior probabilidade de compensação do membro superior por meio do fenômeno de “roubo da subclávia” (1C).
- Lesões da artéria braquial, distais à origem da braquial profunda, podem ser ligadas com baixo risco de isquemia distal (1C)^2, 61, 70^ .

#### Trombose de artéria braquial associada a fratura supracondiliana de úmero com perfusão compensada pode ser tratada de forma conservadora em crianças?

Recomendações:

- Sim. Devido à extensa circulação colateral e à massa muscular proporcionalmente menor do membro superior, quando há uma trombose de artéria braquial com perfusão compensada, a lesão pode ser tratada de forma conservadora, mesmo em crianças, com baixo risco de complicações tardias (1C)^72, 73^ .

#### Nos traumatismos vasculares associados a fraturas, qual a sequência de tratamento mais adequada?

Recomendações:

- No caso de sangramento ativo, a prioridade é interrompê-lo. Fraturas de ossos longos devem, preferencialmente, ser fixadas antes do reparo vascular definitivo. Se a perfusão do membro estiver descompensada, a utilização de um *shunt* vascular temporário deve ser considerada antes da fixação da fratura (1C)^2,60,69^ .

#### Quais critérios/cenários clínicos e escores usar para indicar amputação primária?

Recomendações:

- Escores:
  - Entre os escores já descritos, o *Mangled Extremity Severity Score* (MESS) > 7 é o mais frequentemente utilizado (1C).
  - O *Popliteal Scoring Assessment for Vascular Extremity Injuries in Trauma* (POPSAVEIT) é específico para lesões da artéria poplítea; pacientes com escore 3-5 são considerados de alto risco para amputação (1C).
- Critérios/cenários clínicos:
  - Instabilidade hemodinâmica por hemorragia em extremidade mutilada por múltiplas fraturas expostas, sem possibilidade de controle da hemorragia de outra forma.
  - Apresentação tardia, com ausência de sensibilidade e atividade motora (1C).
  - Perda tecidual extensa e/ou impossibilidade de recuperação funcional (1C).
  - A decisão pela amputação primária é complexa e pode envolver diversas especialidades cirúrgicas, como ortopedia, cirurgia vascular, cirurgia do trauma e cirurgia plástica. Outros escores, além dos citados acima, já foram descritos; todos apresentam limitações e, embora possam auxiliar na tomada de decisão, não substituem o julgamento do cirurgião^2,74^ .

#### Quantas artérias íntegras (radial ou ulnar) no antebraço são suficientes para manter a viabilidade do membro?

Recomendações:

- Normalmente, apenas uma. Se houver perfusão distal compensada por outra artéria, o vaso lesado pode ser ligado (1C)^2,61^ .

#### Quantas artérias íntegras (tibial anterior, posterior ou fibular) na perna são suficientes para manter a viabilidade do membro?

Recomendações:

- A perviedade de duas ou mais artérias reduz a probabilidade de amputações em lesões infrapatelares (1C).
- A integridade de uma artéria tibial anterior ou posterior, com perfusão distal compensada, pode ser suficiente para manter a viabilidade do membro (1C).
  Observação: no contexto dos traumatismos vasculares, não encontramos explicitamente descrito na literatura pesquisada que apenas a integridade da artéria fibular íntegra é suficiente para compensar a perfusão do membro^2,70^ .

#### Qual o papel do tratamento endovascular em lesões de membros?

Recomendações:

- Lesões de artérias subclávia e/ou axilar: tratamento definitivo (stents revestidos) ou adjuvante a cirurgia aberta, através do controle proximal com cateteres de balão (endoclampes) (1C).
- Correção tardia de fístulas arteriovenosas e pseudoaneurismas (1C).
- Embolização de ramos das artérias femorais (especialmente da femoral profunda) e artérias infrapatelares (1C).
- O padrão ouro para traumatismos vasculares das extremidades continua sendo o reparo cirúrgico aberto. O uso de técnicas endovasculares, inclusive em pacientes instáveis, tem sido descrito com frequência crescente, sobretudo para lesões de vasos subclávios e axilares, devido à complexidade dos acessos cirúrgicos a essas estruturas. O cirurgião deve considerar a disponibilidade de todos os recursos, inclusive treinamento da equipe, antes de considerar essa possibilidade (1C) ^2,45, 61^ .

## DISCUSSÃO

Pacientes de todas as idades e gêneros estão sujeitos a sofrerem lesões vasculares traumáticas; entretanto, a vasta maioria das casuísticas aponta o acometimento mais frequente de homens jovens^1-5, 7,18,23,35,45,56^ .

A distribuição das lesões e sua etiologia não são uniformes ao redor o mundo. Enquanto em centros europeus, na Austrália e no Japão, predominam os mecanismos contusos, como acidentes de trânsito; no Brasil, assim como nos centros de trauma americanos, a maior parte das lesões vasculares decorre de traumatismos penetrantes por projéteis de arma de fogo e armas brancas, que acometem com maior frequência os vasos das extremidades^1,17,66,75^ . Além dos mecanismos associados a acidentes e violência urbana, pacientes de todas as idades vêm sendo cada vez mais submetidos a procedimentos diagnósticos e terapêuticos que podem ocasionar traumatismos vasculares iatrogênicos^3,22,48,76^ .

Essas lesões, além de colocarem em risco a viabilidade dos membros, representam uma importante causa de óbito, resultante, na maioria das vezes, de hemorragia não compressível associada a traumatismos dos vasos do tronco^5,41-43,51,52,56-59,61^ .

Ao longo do tempo, novas tecnologias revolucionaram o diagnóstico e o tratamento do trauma vascular. Entretanto, recursos humanos e tecnológicos não estão universalmente distribuídos. A finalidade dessas Diretrizes é ajudar a padronizar e respaldar a conduta adotada para condução de traumatismos vasculares no Brasil, com base em literatura atualizada, que forneça as melhores evidências disponíveis e gere recomendações aplicáveis à realidade assistencial nacional.

A última década testemunhou uma mudança de paradigma no atendimento pré-hospitalar aos traumatizados. Atualmente, o uso de torniquetes para o controle temporário da hemorragia exsanguinante de extremidades acontece em até 20% dos casos, resultando em diminuição importante da mortalidade^2,70^ . Obviamente, o torniquete só se justifica na vigência de hemorragia importante e, assim que possível, deve ser removido para avaliação especializada e, se for o caso, revascularização do membro.

É consenso que pacientes que chegam ao hospital apresentando sinais maiores (*hard signs*) de lesões vasculares traumáticas, sobretudo os que apresentam sangramento ativo e instabilidade hemodinâmica, devem ser submetidos imediatamente a intervenções que visem interromper a hemorragia e, se possível, restaurar a perfusão do membro/órgão afetado^17,19,21,28,35,36^ .

Uma atualização importante diz respeito à condução dos pacientes sem sinais clínicos de lesão vascular dos membros. Embora o exame físico tenha menor sensibilidade para detecção de traumatismos vasculares nos casos de traumatismos contusos de alta energia, como nas luxações de joelho, para os traumatismos penetrantes, que representam o principal mecanismo de trauma vascular das extremidades, as evidências atuais respaldam a alta hospitalar sem a necessidade de investigação com exames de imagem, desde que o paciente apresente exame físico normal (ausência de sinais maiores e menores de lesão vascular) e ITB ≥ 0,9 ^2,6,62,63,69,75^ .

É importante ressaltar que, embora essa estratégia tenha se demonstrado segura, lesões venosas, pseudoaneurismas, fístulas arteriovenosas e outras lesões vasculares menores podem não ser detectadas ao exame físico. A experiência do médico é fundamental e, caso o médico julgue necessário, deve realizar a investigação pertinente. Após a alta, o paciente deve ser encaminhado para avaliações ambulatoriais devido ao risco de complicações tardias de lesões menores incialmente não detectadas^2,63,66^ .

Para todos os cenários que requerem exames de imagem, a angiotomografia se estabeleceu como o atual método padrão ouro para o diagnóstico do trauma vascular^2,35,64,65^ .

Indicações terapêuticas e técnicas de intervenção também evoluíram. Em casos selecionados, algumas lesões podem ser conduzidas de forma não operatória. É o caso, por exemplo, das lesões de artéria braquial associadas a fratura supracondiliana de úmero em crianças, quando a perfusão da mão está compensada - situação atualmente denominada *pink pulseless hand* ^72,73^ . Lesões tamponadas de cava retro-hepática também podem ser passíveis de condução expectante^10,55^ , e critérios para não intervenção em lesões dos membros foram estabelecidos^2,64, 67,70^ .

Os tratamentos endovasculares se estabeleceram em alguns cenários, como nos traumatismos contusos da aorta torácica^15,43,77^ , e, atualmente, vêm sendo empregados com frequência crescente, inclusive em traumatismos penetrantes que requerem acessos complexos, como nas lesões axilo-subclávias^35,37,38,45,78^ .

As estratégias de CD, que incluem os modernos conceitos de ressuscitação, demonstraram-se importantes para o aumento da sobrevida nas lesões mais graves^2,17,52,57,59^ e, por isso, recomendamos treinamento em CD para todos os cirurgiões que possam vir a tratar pacientes com lesões vasculares traumáticas. Algumas das novas estratégias requerem materiais e procedimentos mais complexos, como a oclusão endovascular da aorta^1,4^ ; outras podem salvar membros e vidas com um segmento de uma simples sonda nasogástrica. O *shunt* vascular temporário, ainda pouco frequente nos hospitais brasileiros, já se provou ser uma ferramenta cujo domínio é obrigatório para os cirurgiões que lidam com esses traumatismos^2,17-19,21^ .

Trauma é um campo de estudo diferente de muitas outras condições que afetam o sistema circulatório. Os casos têm mecanismos, gravidade e evoluções heterogêneos, os quais muitas vezes inviabilizam o adequado registro de uma série de variáveis. Esses fatores dificultam escrever diretrizes baseadas em evidências de alta qualidade científica, fazendo com que muitas recomendações sejam baseadas em séries de casos e estudos observacionais.

## CONCLUSÃO

Embora avanços tecnológicos tenham, em parte, revolucionado o diagnóstico e o tratamento do trauma vascular, a condução dessas lesões desafiadoras continua baseada na interrupção precoce do sangramento e na restauração do fluxo sanguíneo para o membro/órgão afetado.

O bom senso e a experiência do cirurgião seguem fundamentais e, embora as recomendações aqui reunidas não os substituam, espera-se que possam ajudar a padronizar e respaldar condutas em casos de trauma vascular, sobretudo considerando as heterogeneidades e peculiaridades assistenciais em nosso país.
